# Projections of winter polynyas and their biophysical impacts in the Ross Sea Antarctica

**DOI:** 10.1007/s00382-023-06951-z

**Published:** 2023-09-23

**Authors:** Alice K. DuVivier, Maria J. Molina, Anna-Lena Deppenmeier, Marika M. Holland, Laura Landrum, Kristen Krumhardt, Stephanie Jenouvrier

**Affiliations:** 1 Climate and Global Dynamics, National Center for Atmospheric Research, Boulder, CO USA; 2 Department of Atmospheric and Oceanic Science, University of Maryland, College Park, MD USA; 3 Woods Hole Oceanographic Institution, Falmouth, MA USA

**Keywords:** Antarctic, Sea ice, Climate change, Model, Polynya

## Abstract

This study investigates winter polynyas in the southern Ross Sea, Antarctica where several polynyas are known to form. Coastal polynyas are areas of lower sea ice concentration and/or thickness along the coast that are otherwise surrounded by more extensive, thicker sea ice pack. Polynyas are also locations where organisms can exploit both the ice substrate and pelagic resources. Using a self organizing map algorithm, we identify polynya events in the Community Earth System Model Version 2 Large Ensemble (CESM2-LE). The neural network algorithm is able to identify polynya events without imposing an ice concentration or thickness threshold, as is often done when identifying polynyas. The CESM2-LE produces a wintertime polynya feature comparable in size and location to the Ross Sea polynya, and during polynya events there are large turbulent heat fluxes and export of sea ice from the Ross Sea. In the CESM2-LE polynya event frequency is projected to decrease sharply in the later twentyfirst century, leading to increasing sea ice concentrations and thicknesses in the region. The drivers of the polynya frequency decline are likely both large scale circulation changes and local atmosphere and ocean feedbacks. If declines in wintertime polynya frequency over the twentyfirst century do occur they may impact Antarctic Bottom Water formation and local net primary productivity. Thus, better understanding potential local and unexpected sea ice changes in the Ross Sea is important for both assessing climate system impacts and ecological impacts on the Ross Sea ecosystem, which is currently protected by an internationally recognized marine protected area.

**Supplementary Information:**

The online version contains supplementary material available at 10.1007/s00382-023-06951-z.

## Introduction

Rising global greenhouse gasses and temperatures have driven large declines in the Arctic sea ice but the Antarctic sea ice response is more nuanced. Studies suggest Antarctic sea ice has declined since the early 1900s (Curran et al 2003; Macalady and Thomas 2017), yet satellite observations since 1979 have shown long term stable or slightly positive trends in total Antarctic sea ice extent (Simmonds 2015). The sea ice trends in different Antarctic regions can vary substantially, be of different sign, and be subject to different variability (Parkinson 2019; Turner et al 2022). Coupled Earth system models have not well captured the pan-Antarctic sea ice trends (Turner et al 2015; Shu et al 2020), but the current generation of models has improved regional sea ice distributions (Roach et al 2020; Casagrande et al 2023). There are also indications that the Antarctic sea ice has large internally generated climate variability, and that this may be particularly important for assessing trends (Roach et al 2020). In recent years there have been precipitous drops in Antarctic sea ice cover (Turner et al 2017, 2022; Raphael and Handcock 2022), and these are likely linked to both atmospheric and oceanic conditions (Stuecker et al 2017; Meehl et al 2019).

Despite having the largest positive trend in regional sea ice cover (Parkinson 2019), the Ross Sea sector of the Antarctic experienced particularly extensive sea ice loss in 2016 and 2022, including the occurrence of an anomalously large coastal polynya (Turner et al 2022). Coastal polynyas—areas of lower sea ice concentration and/or thickness along the coast that are otherwise surrounded by the thicker ice pack—occur all around the Antarctic continent (Arrigo and van Dijken 2003; Tamura et al 2016). Coastal polynyas, also known as latent heat polynyas, are driven by cold downslope winds off the Antarctic continent that push sea ice away from the coast leaving open water that can then freeze into more sea ice (Gordon and Comiso 1988). Polynyas have been deemed ‘sea ice factories’ as they have large sea ice production rates and are where ~ 10% of the total Antarctic sea ice cover is produced and then advected northward, advancing the ice edge (Tamura et al 2008).

The Ross Sea Polynya and Terra Nova Bay Polynya are both important geophysical elements in the Ross Sea regional climate (Petrelli et al 2008). While observations during austral winter are scarce, some observations over the Terra Nova Bay Polynya show exceptionally large turbulent heat fluxes (2500 W/m$^{2}$) from the surface to the atmosphere that vary substantially over relatively small distances (Knuth and Cassano 2014; Ackley et al 2020). Transfer of energy from the open ocean to the atmosphere leads to ocean cooling and eventually high rates of sea ice production (Tamura et al 2016; Schick 2018; Thompson et al 2020). Brine rejection during sea ice formation in polynyas leads to formation of high salinity shelf water, a precursor to Antarctic Bottom Water, the densest water mass in the world oceans (Fusco et al 2009; Kern and Aliani 2011). Thus, Antarctic polynyas, including those in the Ross Sea, impact the global climate through the ocean thermohaline circulation.

Polynyas also impact biology in the Ross Sea as they are in a location where organisms can exploit both the ice substrate and pelagic resources. Polynyas can be the first locations exposed to light in the Antarctic spring and summer, and as a result the Ross Sea has elevated primary production in polynya regions and acts as a carbon sink (Arrigo and van Dijken 2003; Ackley et al 2020). Large phytoplankton blooms in polynya regions support large quantities of krill (Azzali and Kalinowski 2000), and krill are both a source of food for upper trophic levels (McBride 2019) and a focus for commercial fishing (Brooks et al 2019). Polynyas have also been identified as crucial habitat for seals and penguins (Labrousse et al 2018, 2019). Because of the exceptional ecological value of the Ross Sea, in 2016 the international Commission for the Conservation of Antarctic Marine Living Resources (CCAMLR) adopted a 35 year (until 2052) marine protected area (MPA) in the Ross Sea (Brooks et al 2019). The main threats to the biology in the Ross Sea are commercial fishing and climate change, and there is a particular need for studies that integrate ecological processes with physical changes (Brooks and Ainley 2022).

This study addresses the need for biogeophysical studies that provide context for future Ross Sea regional change. We focus on the austral winter (July–August–September) and investigate the following questions:

1. What wintertime sea ice concentration patterns exist in the Ross Sea, and how and why are these patterns changing in time?
2. What are the biogeophysical implications of the winter sea ice cover in the Ross Sea?

We are particularly interested in the austral winter months for this study for three reasons. First, winter polynyas can impact future light and nutrient limitation during the following spring and summer seasons. As regions of lower ice concentration and thickness, winter polynyas have the capacity for preconditioning spring and summer biological productivity as they experience earlier ice retreat than surrounding areas (Mundy and Barber 2001; Arrigo and van Dijken 2003; Arrigo 2007). Additionally, ocean mixing during polynya events may impact nutrient availability. Second, there are important geophysical processes, like formation of dense ocean water, that take place during winter polynya events. Third, the winter wind forcing that drives polynya formation is clear, whereas during the freeze-up or melt seasons clearly differentiating processes that influence polynya formation is more challenging.

To answer our research questions, we use machine learning and coupled earth system model ensemble simulations to investigate polynya changes in the Ross Sea over the century from 1980 to 2079. We then quantify how biogeophysical conditions differ during polynya events and non-polynya events. Finally, we investigate biogeophysical changes from the start to the end of this time period and how that may be related to the changing sea ice conditions.

## Data and methods

### Earth system model experiments

This study uses the Community Earth System Model version 2 (CESM2) (Danabasoglu et al 2020). Antarctic sea ice within CESM2 has been analyzed previously, and was found to have a similar annual cycle and subdecadal variability as compared to observations of Antarctic sea ice (Raphael et al 2020). Additionally, it has similar ice extent and thickness, especially in the Ross Sea, as compared to observations (DuVivier et al 2020). Importantly for this study, CESM2 has more coastal frazil ice formation within polynyas as compared to previous versions of the model (Singh et al 2020). Because we are interested in understanding the forced response of the Ross Sea polynyas, we have decided to use the CESM2 Large Ensemble (CESM2-LE). The CESM2-LE uses standard Coupled Model Intercomparison Project phase 6 (CMIP6) historical forcing (1850–2014) and SSP3$-$7.0 forcing for future experiments (2015–2100) (Rodgers et al 2021). Because the forcing and model are identical to the analyses done in the studies cited above, we expect similarly reasonable Antarctic sea ice properties from the CESM2-LE. To ensure that the CESM2-LE sea ice state in the Ross Sea is reasonable to analyze, we show that the historical (1979–2014) sea ice area in the Ross Sea is comparable between the CESM2-LE and satellite observations. We also calculated the 36 year trends at each latitude, and there are a number of ensemble members that have positive historical trends in the Ross Sea sector, as is seen in the observational record (Supplementary Figure 1). The CESM2-LE ensemble members are all equally likely manifestations of the climate state that differ only due to initial conditions and internal climate variability. Because the CESM2-LE produces some ensembles with positive ice area trends it is therefore capable of capturing the observed trend sign, though it must be noted that no ensemble members have as large of magnitude trends as those observed.

We analyze half (25) of the members of the CESM2-LE that used standard CMIP6 forcing. Each simulation within the CESM2-LE ensemble investigated here use the same model code and forcing. CESM2-LE ensemble members are generated by both macro-perturbations (different ocean state initialization year in 1850) and micro-perturbations (introducing random, round-off level initial atmospheric temperature perturbations (10$^{-14}$ K)). We assume that because all ensemble members use the same forcing they are an equally likely estimate of transient (forced) climate response. The CESM2-LE uses the same standard model configuration that is described in detail in Danabasoglu et al (2020) and Rodgers et al (2021). All components are run at nominal 1${^\circ }$ resolution, and the ice and ocean models share a model grid that has uniform zonal spacing but varying meridional spacing. All model experiments analyzed in this study are freely available to the public (see data availability information at end of manuscript). The CESM2 atmospheric component is Community Atmosphere Model version 6 (CAM6), which has 32 vertical levels, and the ocean component is the Parallel Ocean Program version 2 (POP2), which has 60 vertical levels. CESM2 also includes a prognostic marine biogeochemistry component that simulates multiple phytoplankton and zooplankton functional types (Long et al 2021). CESM2 uses the CICE version 5 thermodynamic-dynamic sea ice model (Hunke et al 2015) with an ice thickness distribution, prognostic sea ice salinity, and salinity dependent freezing temperature (Assur 1958). Compared to previous versions of CESM and other state-of-the-art coupled Earth system models, CESM2 has reasonable simulation of pan-Antarctic sea ice and relatively small biases over the Ross Sea region in both austral summer and winter (Casagrande et al 2023). Additionally, a recent comparison of coupled Earth system models shows that CESM2 has reasonable winter coastal polynya area compared to observations over all of Antarctica (Mohrmann et al 2021). It must be noted that the polynya identification metric used in this study is fundamentally different from that used by Mohrmann et al (2021) as it does not rely on imposed sea ice concentration thresholds and instead identifies times at which there is particularly low sea ice concentration (see following section for details on the machine learning method). Furthermore, this version of CESM employs subgrid-scale calculation of nonlinear biogeochemical functions under sea ice, important for accurate simulation of processes such as phytoplankton photosynthesis (Long et al 2015). Thus, the CESM2 model is a reasonable tool to use to investigate future biogeophysical changes in the Ross Sea related to winter polynyas.

### Self organizing map

This study uses the self organizing map (SOM), a neural network machine learning algorithm to identify patterns of winter sea ice concentration in the Ross Sea and to assess the biogeophysical conditions associated with each type of pattern. The SOM technique is a type of unsupervised learning algorithm that organizes data into groups that are based on similar characteristics without a priori assigned groups of labels. The algorithm uses an iterative learning process that identifies a user-specified number of representative patterns within a dataset (Kohonen 2001; Hewitson and Crane 2002). An advantage of SOMs over other traditional clustering algorithms is that neighboring patterns in a SOM are more similar to each other than those farther apart; thus, SOMs map identified groups to a topological map. The SOM algorithm does not require a priori decisions on data distribution, but it does require the user to choose how many patterns the input dataset will be grouped into. Previous work has compared the SOM method with Principal Component Analysis (PCA) Reusch et al (2005). The methods are fundamentally different but complementary, and the SOM method was better at extracting known patterns from a dataset. While the SOM algorithm doesn’t explicitly identify dominant patterns, analysis of pattern frequencies can provide insight into which patterns occur most often. We chose the SOM method because we seek to identify and represent sea ice patterns from the model that are analogous to what might be observed satellite observations of sea ice concentration. We can then investigate processes associated with these types of spatial patterns. Detailed information on the SOM method and about choosing SOM patterns for Earth science can be found in Kohonen (2001) and Cassano et al (2015), but we provide a short description below with relevant information for this study.

**Fig. 1:**
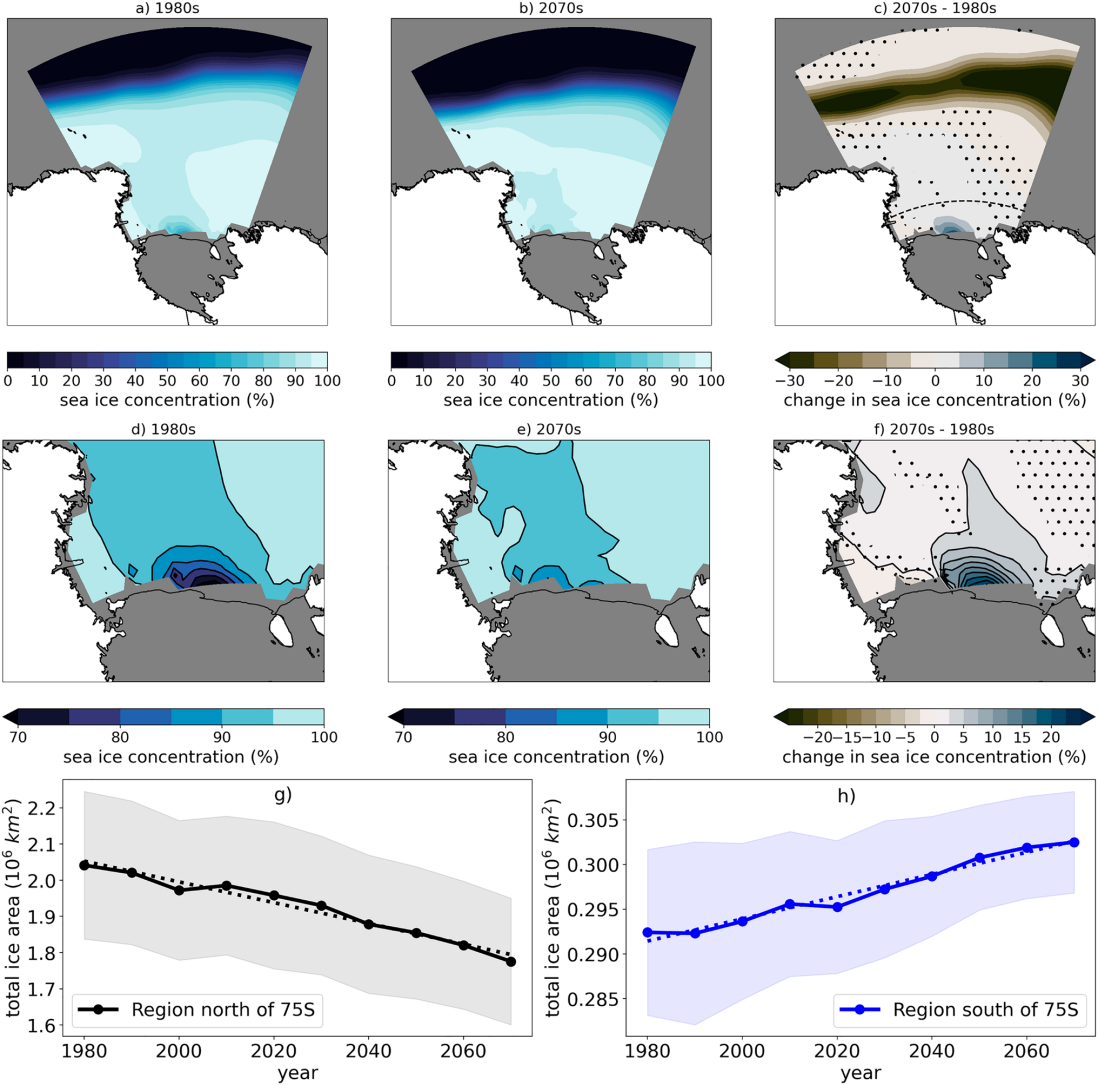
Winter sea ice concentration and trends in the Ross Sea, Antarctica. CESM2-LE 25 member ensemble mean sea ice concentration (%) in the **a** 1980s, **b** 2070s, and **c** difference (2070s–1980s). (**d**–**f**) are identical to (**a**–**c**), except zoomed in on the Southern Ross Sea. The dashed line on (**c**) is the 75$^\circ$ S latitude line and stippling on (**c**) and (**f**) indicates that differences are NOT significant at the 95% confidence level using a two-tailed 10,000 member bootstrap re-sampling test at each grid point (Chernick 2011). The wintertime total sea ice area by decade (circles) and trends (dashed lines) is shown for the Ross Sea sector **g** north of 75$^\circ$ S latitude and **h** south of 75$^\circ$ S latitude; shading indicates one standard deviation around the ensemble mean

The SOM was trained using the MiniSOM python package (Vettigli 2018). We used sea ice concentration from 189 coastal grid cells in the Ross Sea (Supplementary Figure 2) where there are projected increases in austral wintertime sea ice concentration in future decades (Fig. 1). The training data are 1980–2079 winter (July, August, September) daily average sea ice concentration from 25 members of the CESM2-LE (232,300 total training dates). During training, each of the training dates is mapped to the representative pattern it best matches. These lists of training dates associated with each node allow us to both construct composite averages of each pattern and to calculate the frequency with which a pattern is represented within a given year, decade, or the entire dataset.

To train the SOM, we tested SOMs with various numbers of patterns and different values of training hyperparameters for: 1) the spread of the neighborhood function, 2) the number of training iterations, and 3) the initial learning rate. The spread of the neighborhood function (sigma) determines how many surrounding patterns around the best matching pattern to the respective training sample gets updated as the SOM training progresses. The number of iterations is how many times the training samples are presented to the SOM algorithm. The initial learning rate determines how strongly patterns are nudged toward the training data, but this rate and sigma decrease to zero during the iterative training process at a rate determined by an asymptotic decay function. Asymptotic decay of the learning rate and sigma ensures that neighboring patterns have similarities within a SOM, but are sufficiently different to be independent groups. Further details about these training hyperparameters are detailed in Vettigli (2018). We trained a total of 360 SOMs, both testing a range of hyperparameters and testing for identification using a 3 $\times$ 3 size (9 patterns, i.e., nodes), 4 $\times$ 4 size (16 patterns), and 5 $\times$ 5 size (25 patterns). Jupyter notebooks of the training are freely available for those wishing to replicate the results (https://github.com/duvivier/CESM2_Ross_Sea_polynya_analysis).

The ‘winning’ SOM that we analyze for the rest of the manuscript was chosen based on the following criteria: (1) a low quantization error (qerror), (2) a flat (i.e. not “twisted”) Sammon mapping of the identified patterns indicating smooth pattern topology, and (3) physically meaningful patterns based on domain expertise. The qerror and Sammon mapping for the SOM used in this manuscript are shown in Supplementary Figure 2. The qerror is an output of the SOM software and is computed as the distance between each training sample and its closest node. Having a lower qerror indicates a better fit between the training data and the patterns identified. The Sammon map is a data dimensionality reduction method and 2D visualization of the patterns identified by the SOM, in which the linear distance between patterns signifies the similarity between patterns. In a flat Sammon map, adjacent patterns are more similar to one another than patterns relatively far apart and the relationship between the patterns is clear, but sometimes the map can be “twisted” and then the relationship between adjacent patterns is not readily interpretable. Finally, we chose a SOM in which there were patterns that look similar to those one might see by looking at satellite data of sea ice concentration, and therefore the patterns are physically meaningful for analysis.

## Results

Using an ensemble mean from 25 members of the CESM2-Large Ensemble (Rodgers et al 2021), we find that in the Ross Sea sector of the Antarctic, there are contrasting winter sea ice concentration trends north and south of 75$^{\circ }$ S latitude (Fig. 1). Sea ice cover is being lost along the northern ice edge at a rate of $-\,2.87 \times 10^{2}$ million km$^{2}$ per decade ($r^{2} = 0.97$), while near the Ross Ice Shelf sea ice cover is increasing at a rate of $+\,0.12 \times 10^{2}$ million km$^{2}$ per decade ($r^{2} = 0.97$). The trend in projected ice cover increases in the southern Ross Sea is consistent over the century from 1980 to 2079. The following analysis focuses on the Southern Ross Sea, south of 75$^{\circ }$ S latitude where the future ice concentration increases are concentrated at the edge of the Ross Ice Shelf and become more pronounced by decade (Supplementary Figure 3). By the 2070s, along the Ross Ice Shelf the mean winter sea ice concentration is more than 20% higher than it was in the 1980s (Fig. 1d–f).

### Winter sea ice patterns

**Fig. 2:**
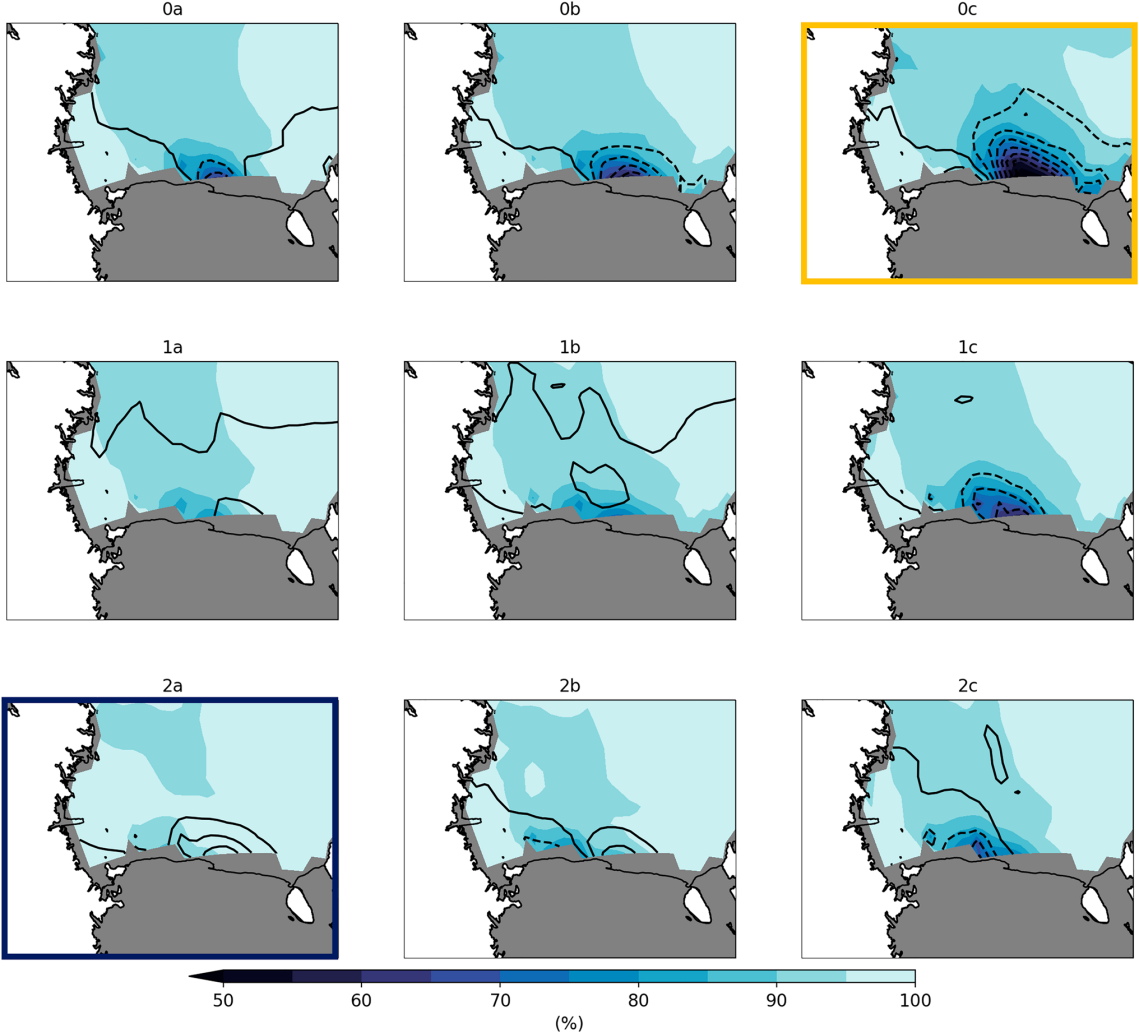
Composite sea ice concentration for nine patterns identified by the SOM. Sea ice concentration (%) for each pattern is calculated by averaging each of the training dates from the 25 CESM2-LE members using daily winter sea ice concentration from 1980 to 2070. Black contours show the ice concentration anomaly (%) for that pattern from the wintertime mean ice concentration. Pattern 0c (gold border) and 2a (dark blue border) indicate the patterns that are analyzed in detail

The composite winter sea ice concentration patterns in the southern Ross Sea that are identified by the SOM are shown in Fig. 2. The SOM is arranged such that in the upper right are patterns with a polynya feature at the edge of the Ross Ice Shelf while patterns with full ice cover and no polynya are in the lower left of the SOM. The patterns with the largest differences from one another are in the corners of the SOM. Pattern 0c has the largest polynya and concentration anomalies of − 30% compared to the wintertime mean. Patterns 0b and 1c also have polynyas, though smaller in extent with slight differences in the location and extent of the polynya. In contrast, Pattern 2a contains anomalous ice concentrations near the Ross Ice Shelf of + 15% and the entire Ross Sea is ice covered. For the following analysis we will compare polynya cases (Pattern 0c—gold border on Fig. 2) and non-polynya cases (Pattern 2a—dark blue border on Fig. 2) to highlight the biogeophysical differences that occur during the winter with different sea ice conditions. However, the full SOM with all nine patterns for each of the variables discussed below is provided in Supplementary Figures 4–16.

**Fig. 3:**
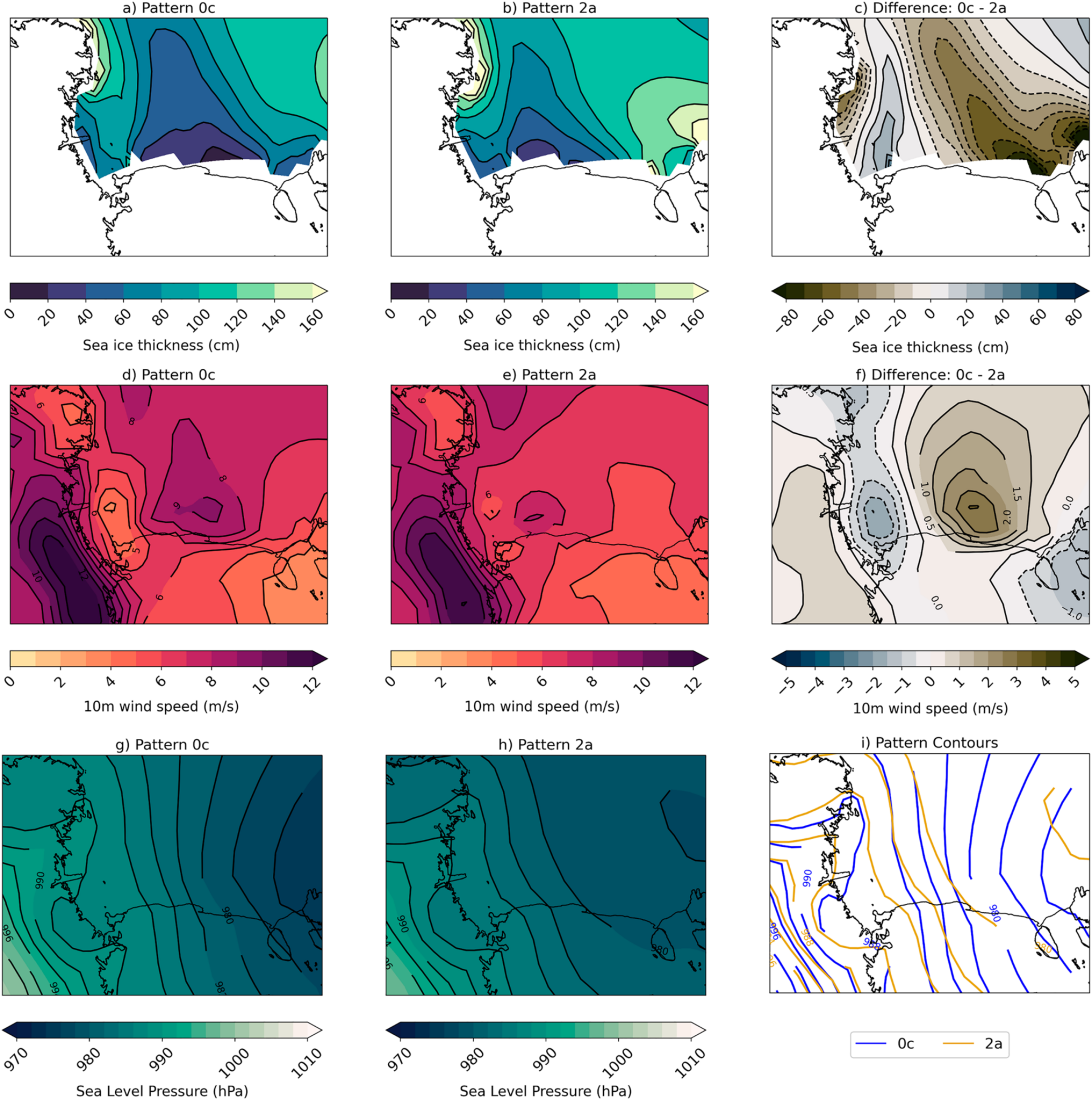
Composite sea ice and atmospheric conditions for SOM patterns of interest. Sea ice thickness (cm) for **a** Pattern 0c, **b** Pattern 2a, and **c** Difference; 10 m wind speed (m/s) for **d** Pattern 0c, **e** Pattern 2a, and **f** Difference; sea level pressure (hPa) for **g** Pattern 0c, **h** Pattern 2a, **i** Difference. All differences are calculated as follows: Diff = (Pattern 0c − Pattern 2a)

A tongue of relatively thinner sea ice, as compared to the rest of the Ross Sea, extends from the Ross Ice Shelf in both polynya and non-polynya events, but the sea ice in this tongue is up to 70% (40–60 cm) thinner during polynya events (Fig. 3a–c). In addition, during polynya events there is a near-surface wind jet that originates at the edge of the Ross Ice Shelf and this increases the near surface wind speeds by up to 30% (3 m/s) (Fig. 3d–f). The wind direction is southerly/southeasterly in both patterns, but the sea level pressure gradient is stronger during polynya events and would drive increased geostrophic wind speeds (Fig. 3g–i).

**Fig. 4:**
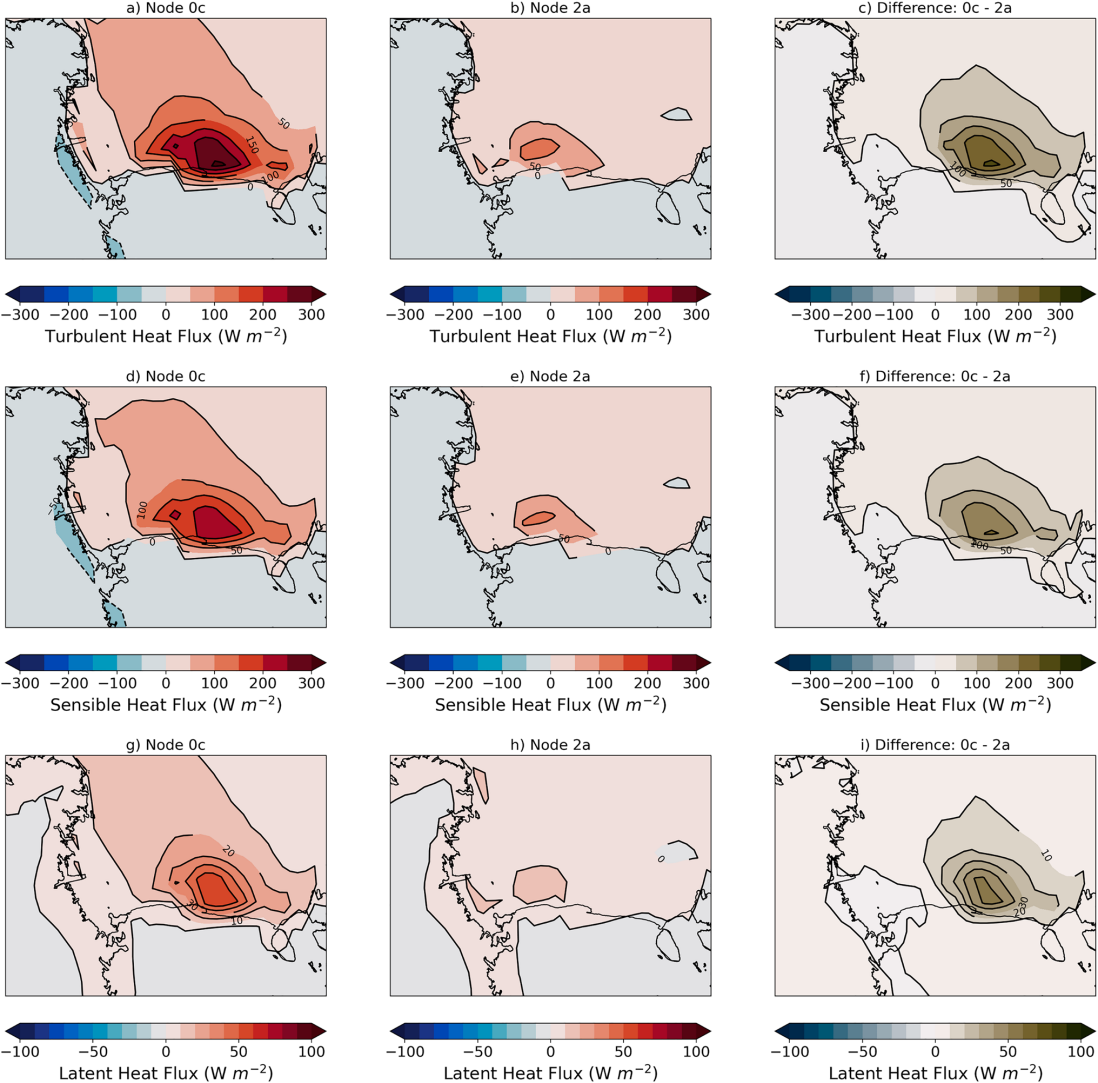
Composite turbulent fluxes to the atmosphere for SOM patterns of interest. Total turbulent heat flux (W/m$^2$) for **a** Pattern 0c, **b** Pattern 2a, and **c** difference; sensible heat flux (W/m$^2$) for **d** Pattern 0c, **e** Pattern 2a, and **f** difference; latent heat flux (W/m$^2$) for **g** Pattern 0c, **h** Pattern 2a, and **i** difference. All differences are calculated as follows: Diff = (Pattern 0c − Pattern 2a). Positive (negative) heat flux values indicate atmospheric energy gain (loss)

**Fig. 5:**
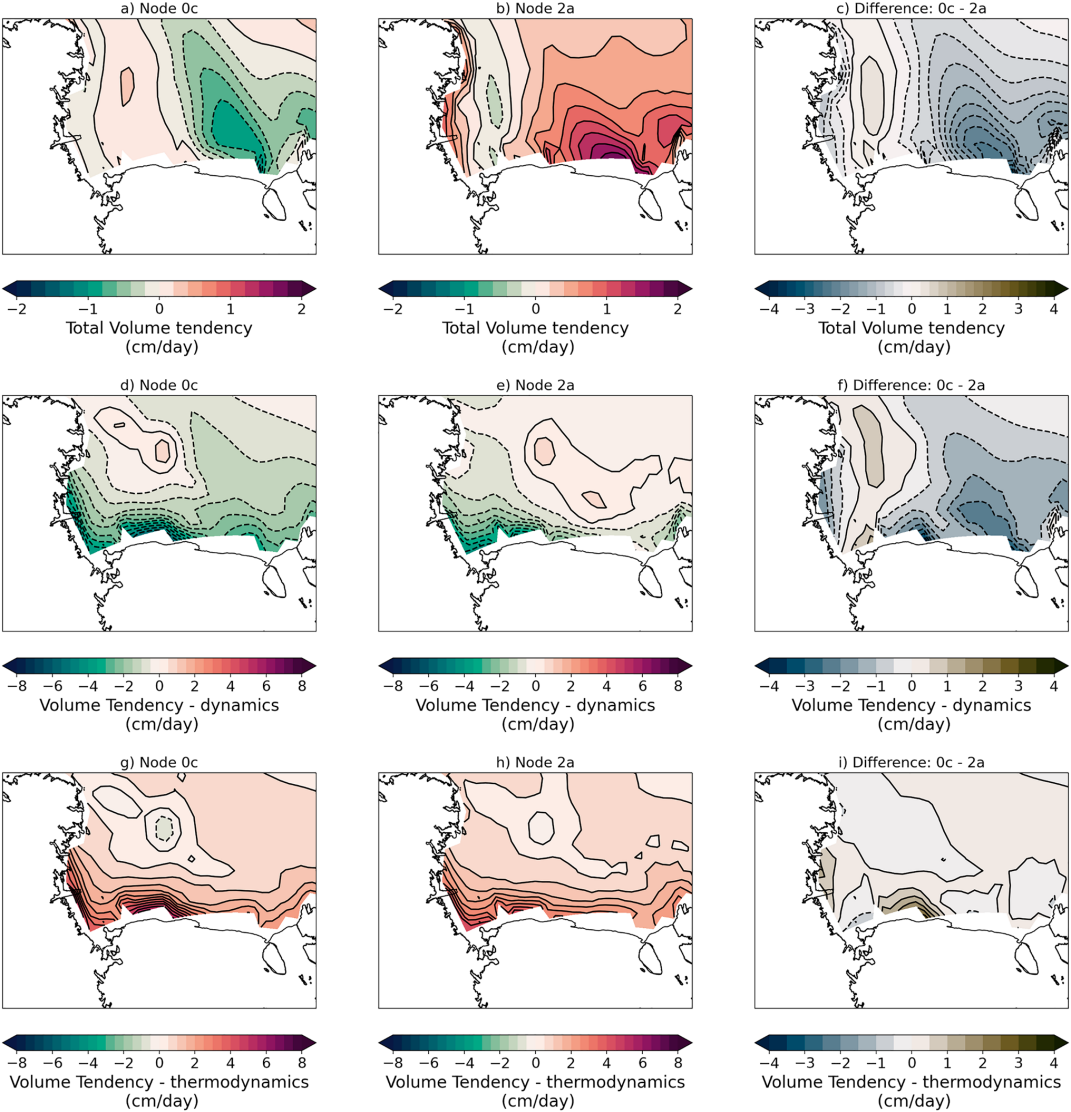
Composite sea ice volume tendencies for SOM patterns of interest. Total sea ice volume tendency (cm/day) for **a** Pattern 0c, **b** Pattern 2a, and **c** difference; dynamics contribution to sea ice volume tendency (cm/day) for **d** Pattern 0c, **e** Pattern 2a, and **f** difference; thermodynamics contribution to sea ice volume tendency (cm/day) for **g** Pattern 0c, **h** Pattern 2a, and **i** difference. All differences are calculated as follows: Diff = (Pattern 0c − Pattern 2a). Positive (negative) ice tendency values indicate ice gain (loss)

As a result of the less extensive, thinner ice and faster winds during polynya events there are significantly higher turbulent heat fluxes of energy to the atmosphere (Fig. 4). During polynya events there are up to 250 W/m$^2$ more energy transfer from the ocean to the atmosphere over the polynya, most of which is primarily driven by the sensible heat flux component (up to 200 W/m$^2$). In addition to having higher fluxes during the polynya events, the peak fluxes occur further east along the Ross Ice Shelf compared to during non-polynya events. As seen in Fig. 5a–c, the sea ice volume tendencies over the southern Ross Sea differ by up to 2.5 cm/day during polynya vs. non-polynya events with polynya events losing ice on the whole (− 1.08 km$^3$/day south of 75$^\circ$ S latitude) while non-polynya events gain ice on the whole (+ 2.16 km$^3$/day south of 75$^\circ$ S latitude). Both polynya and non-polynya events have negative dynamic contributions to the volume tendency (meaning net ice volume transport from the region), though the total ice loss south of 75$^\circ$ S latitude is more than two times higher in polynya events (− 6.54 km$^3$/day) compared to non-polynya events (− 2.91 km$^3$/day). In contrast, the thermodynamic contribution to the volume tendency and total ice growth south of 75$^\circ$ S latitude is relatively similar for both polynya events (+ 5.46 km$^3$/day) and non-polynya events (+ 5.07 km$^3$/day). Given the large differences in turbulent heat fluxes to the atmosphere during polynya and non polynya events (Fig. 4), the relatively small difference in total volume tendency from thermodynamics is unexpected and suggests the turbulent atmospheric fluxes are not the only factor affecting the thermodynamic ice volume tendency, but ocean processes are impacting the ice volume as well.

**Fig. 6:**
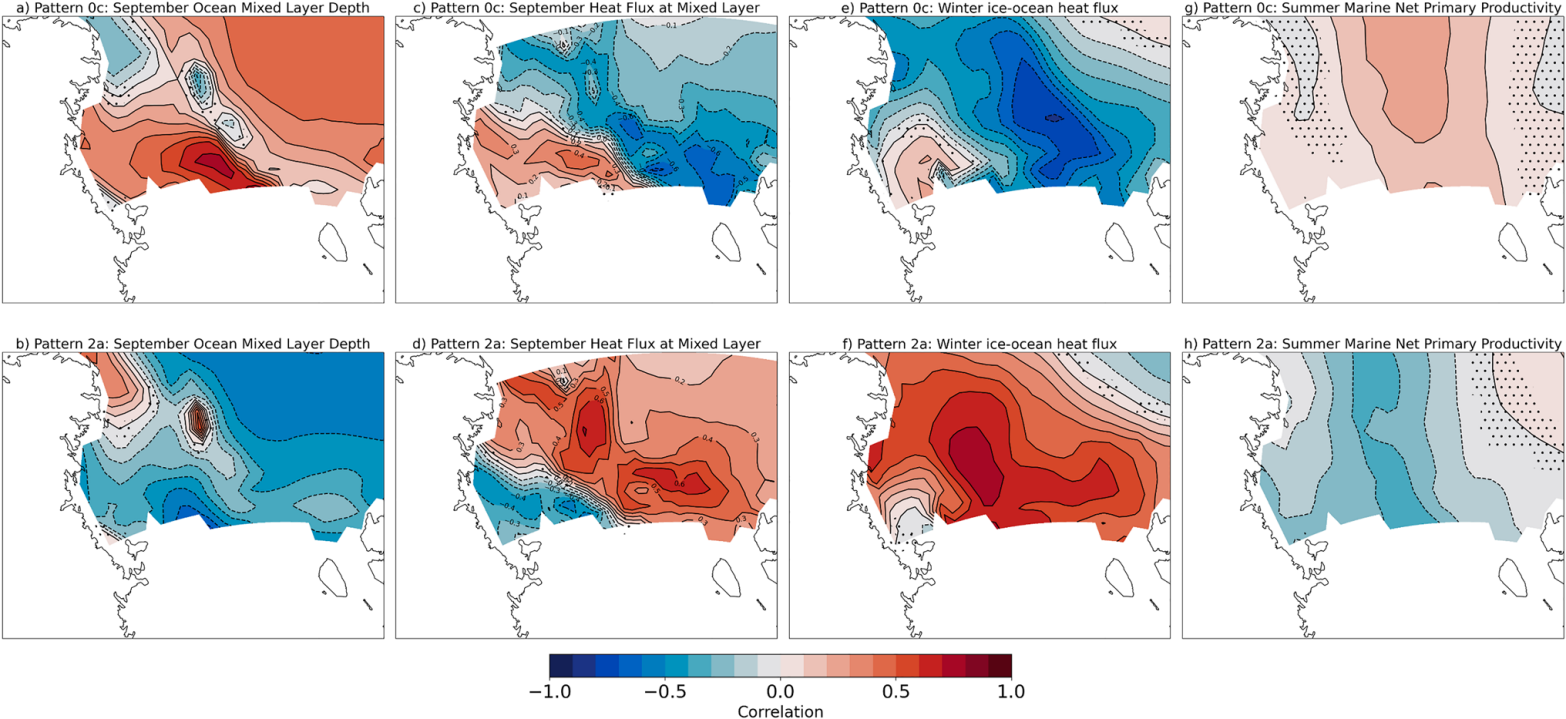
Correlation of Ocean variables with frequencies of SOM patterns of interest. Correlation of end of winter mixed layer depth with frequency of **a** Pattern 0c and **b** Pattern 2a; correlation of end of winter heat flux through the mixed layer with frequency of **c** Pattern 0c and **d** Pattern 2a; correlation of end of winter mean ice–ocean heat flux with frequency of **e** Pattern 0c and **f** Pattern 2a; correlation of end of winter heat flux through the mixed layer with frequency of **g** Pattern 0c and **h** Pattern 2a. Stippling indicates points at which the correlation is NOT significant at a 95% confidence level using a 2-tailed *p* value associated with Pearson’s correlation coefficient. Negative heat flux through the mixed layer indicates an upward flux. Negative ice–ocean heat flux indicates ocean heat loss to the ice, usually due to sea ice melt

The physical process differences during polynya and non-polynya events impact the Ross Sea both physically and biologically. Because ocean output from the CESM2-LE is limited temporally, we are unable to create composite averages as shown before. Therefore, to investigate how polynyas comparatively drive ocean mixing and heat transport, we correlate the frequency of Patterns 0c (polynya event) and 2a (no polynya) with the end of winter mixed layer depth, the end of winter heat flux through the mixed layer, and the winter average ice–ocean heat flux. A negative heat flux at the mixed layer indicates an upward heat flux, or heating of the mixed layer from below. Positive (negative) ice–ocean fluxes indicated ocean heat gain (loss) to the ice, generally due to ice growth (melt). We find that for Pattern 0c there is a positive, significant correlation with ocean mixed layer depth (Fig. 6a). Thus, when there is a higher frequency of polynya events the mixed layers are deeper. Deeper mixed layers entrain warmer water at depth, which inhibits ice growth or melts sea ice during polynya events where there are large turbulent heat fluxes from the surface to the atmosphere, as described in the previous paragraph. We find that for Pattern 0c there is a significant negative correlation with the heat flux through the mixed layer over much of the Ross Sea (Fig. 6c). A negative correlation indicates that when there are more frequent polynya events the heat flux is more negative, or that there is more heating of the mixed layer from below. The significant negative correlations of Pattern 0c with the ice–ocean heat flux indicate that when there are more frequent wintertime polynya events there are higher ice–ocean heat fluxes into the sea ice that could melt ice (Fig. 6e). Opposite correlations are found for Pattern 2a (no polynya), indicating that when the Ross Sea is ice covered more often then there are shallower mixed layers, less entrainment of heat from depth, and less flux from the ocean to melt sea ice. This reinforces the no-polynya pattern. The relationship between polynyas, ocean mixing, and ice production also has implications for Antarctic Bottom Water (AABW) production, which will impact the global thermohaline circulation as AABW is the densest water mass in the global ocean; these relationships are important, but will not be investigated further in this study. Additionally, deeper mixed layers can contribute to more nutrients being mixed to the surface. We correlated the marine net primary production (NPP) the following summer with frequency of Patterns 0c and 2a the previous winter. We find significant positive, though fairly weak, correlations over nearly the whole Ross Sea (Fig. 6g), which indicates that there is a weak connection between more frequent wintertime polynya events and increased NPP the following summer.

### Mechanisms driving decadal differences

We also use the SOM to untangle why the mean decadal difference in sea ice concentration exists. We focus on the difference between the 1980s and 2070s because the sea ice concentration differences are largest (see Fig. 1), but similar analysis was performed on other decades with consistent results to those presented here.

In particular, we investigate if the decadal sea ice changes are related to changes in SOM pattern frequency, pattern representation, or the combination of these two. For a given decade, we can calculate the decadal mean by averaging over all days in the winter, all years in the decade, and all 25 ensemble members used in this analysis. We can also calculate the decadal mean using the following equation:

1 $$\begin{aligned} \ {SIC_{mean} = \sum _{p=1}^{P} [f_{p}} * SIC_{p}], \end{aligned}$$

where $f_p$ is the pattern frequency and $SIC_p$ is the sea ice concentration for each of the nine patterns (P = 9) identified by the SOM, calculated using only the training data for the decade of interest. Following previous studies (e.g. Cassano et al 2007; DuVivier and Cassano 2016), we calculate the decadal difference between the 1980s and 2070s using the following equation:

2 $$\begin{aligned} \ {SIC_{diff} = \sum _{p=1}^{P} [(\Delta f_{p}} * SIC_{1980,p}) + (f_{1980,p} * \Delta SIC_{p}) + (\Delta f_{p} * \Delta SIC_{p}) ], \end{aligned}$$

where $\Delta f_{p}$ is the difference in frequency between decades ($\Delta f_{p} = f_{1980,p} - f_{2070,p}$) for a given pattern and $\Delta SIC_{p}$ is the difference in the composite pattern between decades ($\Delta SIC_{p} = SIC_{1980,p} - SIC_{2070,p}$) for a given pattern. In Eq. 2, the sum of just the first term ($\sum _{p=1}^{P} \Delta f_{p} * SIC_{1980,p}$) is the frequency contribution to the total difference between decades due to changes in how often the training data from the two decades are assigned to the same pattern. The sum of just the second term ($\sum _{p=1}^{P} f_{1980,p} * \Delta SIC_{p}$) is the pattern contribution to the total difference between decades due to differences in how a particular pattern is represented in training data from both decades. Finally, the sum of just the last term ($\sum _{p=1}^{P} \Delta f_{p} * \Delta SIC_{p}$) is the contribution to the mean difference due to changes in frequency and changes in pattern representation working together.

**Fig. 7:**
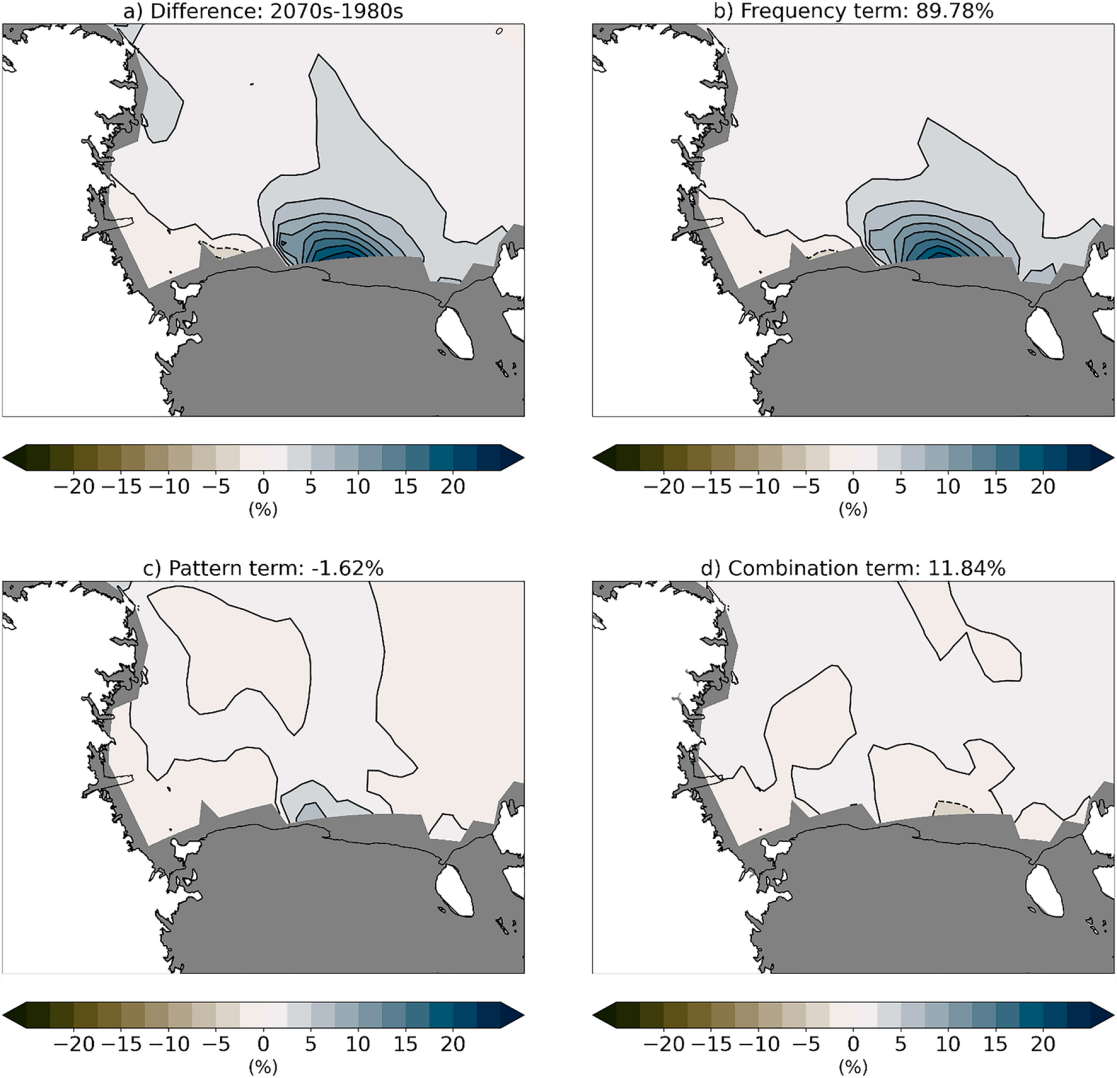
Contribution of the frequency, pattern, and combination terms to the decadal 2070–1980 sea ice concentration difference. Terms are calculated as shown in Eq. 2, where **a** is the total decadal difference, **b** is the frequency term, **c** is the pattern term, and **d** is the combination term. Percentages listed in the panel labels indicate the mean contribution of that pattern to the total difference shown in (**a**)

**Fig. 8:**
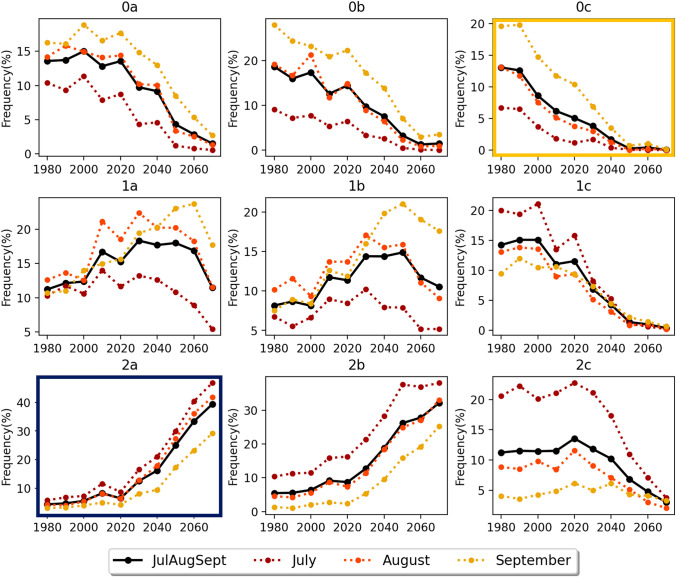
Decadal frequency for nine patterns identified by the SOM. Frequencies (%) are calculated for all winter (black), July (dark red), August (orange), and September (gold). Pattern 0c (gold border) and 2a (dark blue border) indicate the patterns that are analyzed in detail

Using Eq. 2, we calculate the total difference as well as the frequency, pattern, and combined contributions. As expected, the total difference (Fig. 7a) matches the difference previously shown in Fig. 1f, but that was calculated by a traditional average. We find that the frequency term has the largest contribution (90%) to the total decadal sea ice concentration difference between the 1980s and 2070s. When we examine the decadal frequency differences (Fig. 8) the reason for this dominance is clear. Patterns with different types of polynyas (see Fig. 2, Patterns 0a, 0b, 0c, 1c, 2c) all have decreasing frequency in future decades over all winter months. In contrast, patterns with more extensive Ross Sea ice cover (see Fig. 2, Patterns 2a, 2b) have consistent increases in frequency over future decades and over all winter months. The CESM2-LE shows that polynyas of any kind become rare in the future. Each winter month experiences a decline, which indicates there is not a change in seasonality of polynya events throughout the winter months. There are also not systematic declines in polynya size, which would be reflected by a decrease in large polynya patterns like 0c but an increase in adjacent patterns with smaller polynyas.

**Fig. 9:**
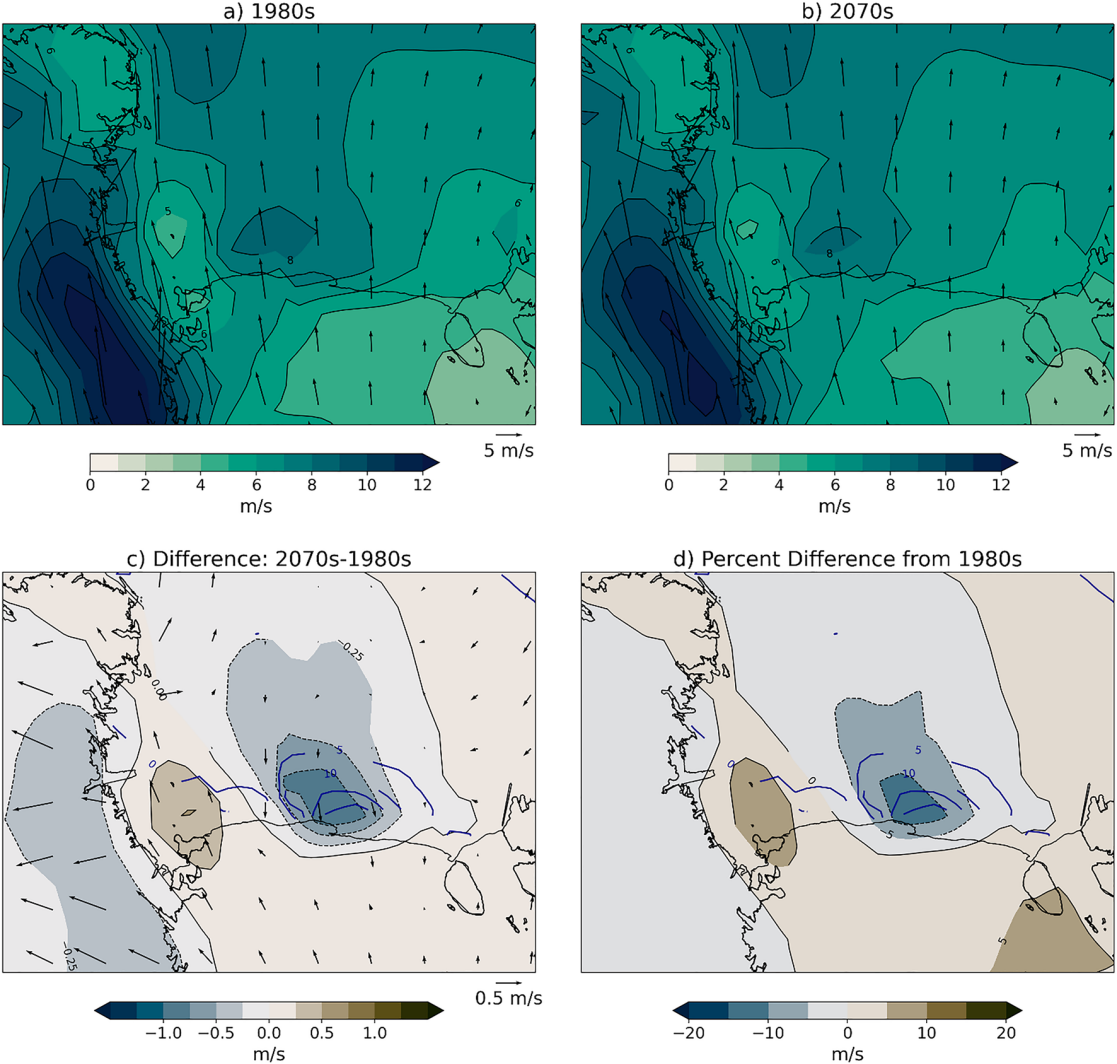
Mean winter wind field and difference 1980s and 2070s. Wind speed (m/s) (shading) and wind vectors for **a** 1980s, **b** 2070s, **c** difference (2070s–1980s), and **d** percent difference of scalar wind speed. The dark blue contours shown on (**c**) and (**d**) are the decadal sea ice differences from Fig. 2c at a 5% interval

Next, we examine how shifts in mechanisms may cause the decrease in frequency of polynya patterns. We focus on the wind forcing in the Ross Sea because the Ross Sea Polynya is forced by southerly winds coming off the Antarctic continent. The mean winter wind field in both the 1980s and 2070s is southerly winds from over the Ross Ice Shelf. In the 2070s there is not a significant change in wind direction over the Ross Sea, but the wind speed is about 10% slower in the 2070s compared to the 1980s directly over where there are increases in sea ice concentration (Fig. 9). We performed 95% level significance tests with two-tailed 10,000 member bootstrap re-sampling test at each grid point (Chernick 2011) and found that that majority of points were not significantly different. We have investigated three factors that could contribute to this decrease in near-surface wind speed.

**Fig. 10:**
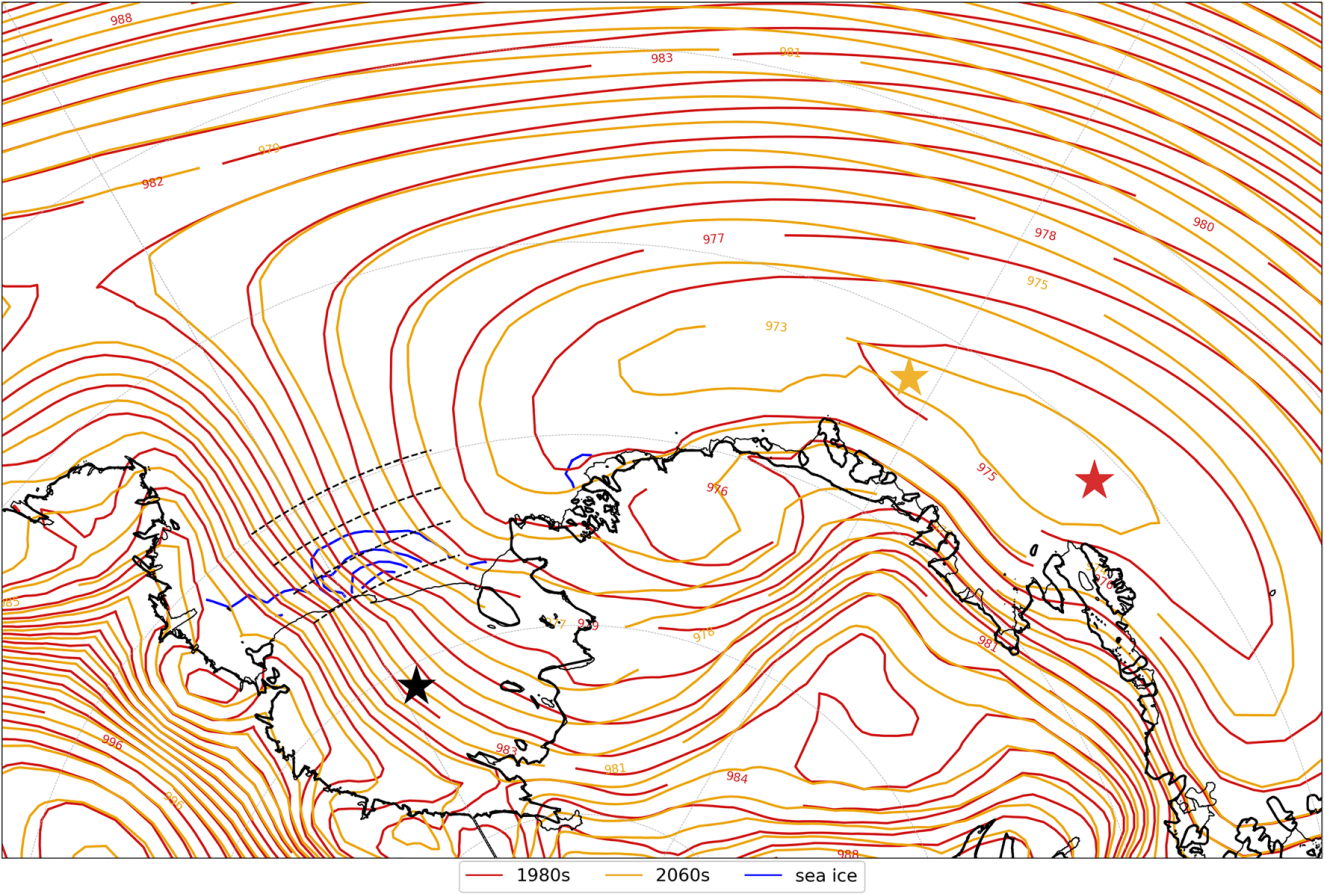
Mean winter sea level pressure 1980s and 2070s. Red contours indicate the 1980s and the red star marks the location of the low pressure center; gold contours indicate the 2070s and the gold star marks the location of the low pressure center. The black star indicates the location of the Ross Ice Shelf, and the blue contours in the Ross Sea show the decadal sea ice differences from Fig. 2c at a 5% interval. The dashed black lines over the Ross Sea indicate the lines of constant latitude along which pressure gradients were calculated

The first is changing geostrophic wind speed due to changes in large scale circulation. We find that in CESM2-LE the wintertime Amundsen Sea Low (ASL) deepens and shifts westward (toward the Ross Sea) in the 2070s (Fig. 10), which results in a small decrease in the local pressure gradient and weakening of the winds over the Ross Sea polynya (Fig. 9). The deepening of the ASL is consistent with observations (Raphael et al 2016), other CESM2 analyses (Drijfhout 2022), and analysis of CMIP5 models (Hosking et al 2016). The mean wintertime response of the ASL across CMIP5 models is an eastward shift with non-statistically significant differences near the Ross Sea ice shelf (Hosking et al 2016). The differences in the ASL response to climate change in different climate models indicate there is uncertainty in how the Ross Sea Polynya will respond to a changing ASL in the future. Yet this study shows that small location shifts in the position of the ASL drive differences in the off-shelf wind speed (though not direction) in the Ross Sea, and better understanding the small wind shifts that can trigger climate feedbacks is important for fully projecting future changes. It is also important to note that mesocyclone activity, which is relevant for sea ice growth and high salinity shelf water production (Wang et al 2023), is not well captured in the relatively low resolution models used here and in (Hosking et al 2016), but better resolving local atmospheric flow and cyclones is likely to strongly impact future polynya behavior in the Ross Sea. For this study, we are particularly interested in how the shift in the large scale circulation may affect local winds in the Ross Sea. We calculated the pressure gradient, which is proportional to wind speed, over four lines of latitude across the Ross Sea where the largest ice concentration differences are and found that there were decreases in the pressure gradient by about 5% in the 2070s as compared to the 1980s that would contribute to decreases in wind speed.

**Fig. 11:**
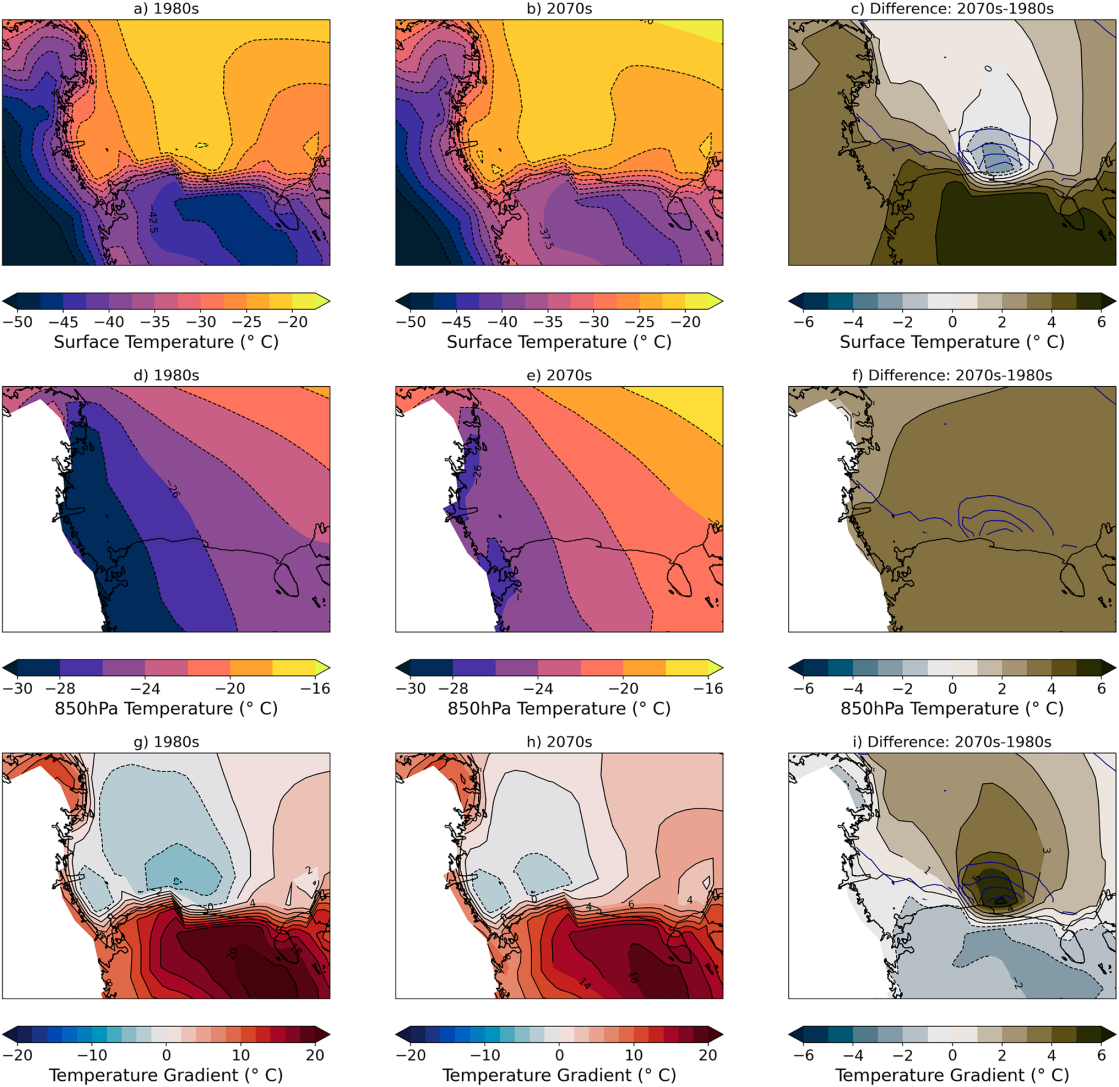
Mean winter temperature and difference 1980s and 2070s. Surface temperature ($^{\circ }$C) for **a** 1980s, **b** 2070s, and **c** difference; 850 hPa temperature ($^{\circ }$C) for **d** 1980s, **e** 2070s, and **f** difference; temperature gradient (T$_{850hPa}$ − T$_{sfc}$; $^{\circ }$C) for **g** 1980s, **h** 2070s, and **i** difference. All differences are calculated as follows: Diff = (2070s–1980s). A positive (negative) gradient indicates (stable) unstable conditions. In (**c**), (**f**), and (**i**) the dark blue contours are the decadal sea ice differences from Fig. 2c at a 5% interval

The second factor that could contribute to the decrease in wind speed is the near-surface stability, which could impact turbulent mixing of stronger winds aloft toward the surface. We use the temperature difference between 850 hPa and the surface (T$_{850hPa}$ − T$_{sfc}$) as a proxy for stability; thus a positive (negative) gradient indicates stable (unstable) conditions and a positive difference in the gradient indicates that conditions are becoming more stable. We find that at the surface there is a small cooling of 1–2 $^{\circ }$C in the same location where sea ice concentration increases while there is warming nearly everywhere else in the Ross Sea (Fig. 11c); despite some locations warming, the winter surface temperatures are still cold enough (below $-20\,^{\circ }$C) to drive sea ice formation. At 850 hPa there is a nearly constant increase in temperature of around 3–4 $^{\circ }$C (Fig. 11f). These changes at the surface and aloft result in increasing stability in the same region where there are increases in sea ice cover and decreases in wind speed (Fig. 11i). These increases in stability imply decreased mixing of stronger winds above the surface downward. The third possible impact on the near surface winds are the changes in surface roughness related to sea ice cover. In the CESM2-LE model experiments, sea ice is rougher than over the open ocean. Thus, the surface roughness that the atmosphere experiences in the 2070s will be higher than in the 1980s, and this will increase surface-atmosphere drag and could lead to decreases in wind speed.

**Fig. 12:**
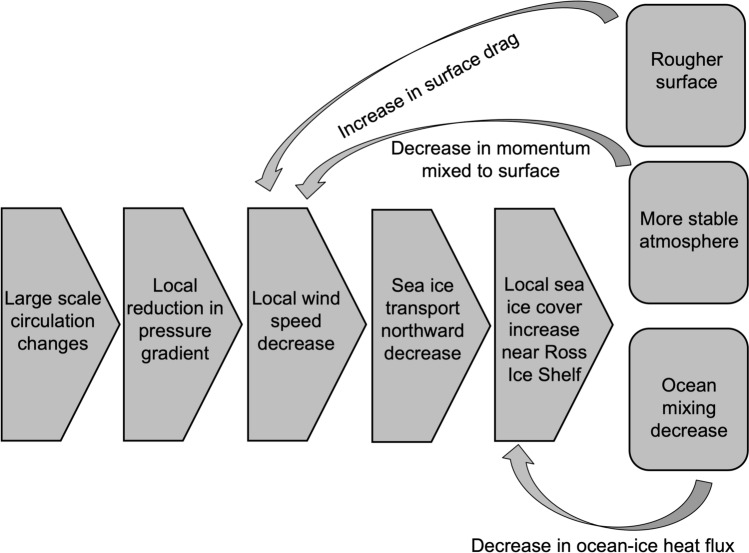
Proposed mechanism and feedback processes for future wind and sea ice changes

As shown in Fig. 12, we hypothesize that the decreases in sea ice cover in the 2070s in the Ross Sea are due to a combination of atmospheric and oceanic feedbacks. The small changes in the ASL and large scale circulation likely initiate a small, local reduction in the pressure gradient that would lead to wind speed decreases, which then lead to less sea ice transport northward, out of the Southern Ross Sea. The local increases in sea ice cover result in both a rougher surface and a decrease in the surface temperature that causes the atmospheric stability to increase, both of which then have a positive feedback to further decrease local wind speeds and cause local sea ice cover to increase. In addition to atmospheric feedbacks, the high correlation between polynya events and ocean mixing suggests that ocean feedbacks may also contribute to increasing Ross Sea ice concentrations. With decreasing wind speeds and fewer polynya events, ocean mixing would be shallower and the ocean-to-ice heat flux that could inhibit sea ice growth would also decrease. As a result of the combination of these mechanisms, polynya events in CESM2-LE become rare in future decades rather than a frequent winter occurrence.

It is important to note that the decreases in wind speed are localized to the Ross Sea and that around much of Antarctica there are projected increases in wind speed likely related to deeper lows around the Antarctic continent and increasing high pressures farther north. The projected changes in the large scale circulation in CESM2-LE result in a positive trend in the Southern Annular Mode (SAM) index (Supplementary Figure 17), which would then lead to a southward shift of the westerlies that ring the continent. Thus, the projected local changes in polynyas, sea ice, and wind speed in the Ross Sea are very much an outlier in the context of the entire Antarctic continent.

### Future biogeophysical implications

This analysis was initiated in order to understand the causes and implications of projected increases in winter sea ice concentration in the Ross Sea in future decades (Fig. 1). As shown in Sect. 3.2, there are more polynya events in the 1980s than the 2070s, and the event frequency shift drives the mean winter difference in sea ice state. Large differences in biogeophysical fields during polynya and non-polynya events, as described in Sect. 3.1, imply that it is likely there may be significant biogeophysical climate implications in the decadal means as the conditions from polynya or non-polynya events accumulate over a winter season.

**Fig. 13:**
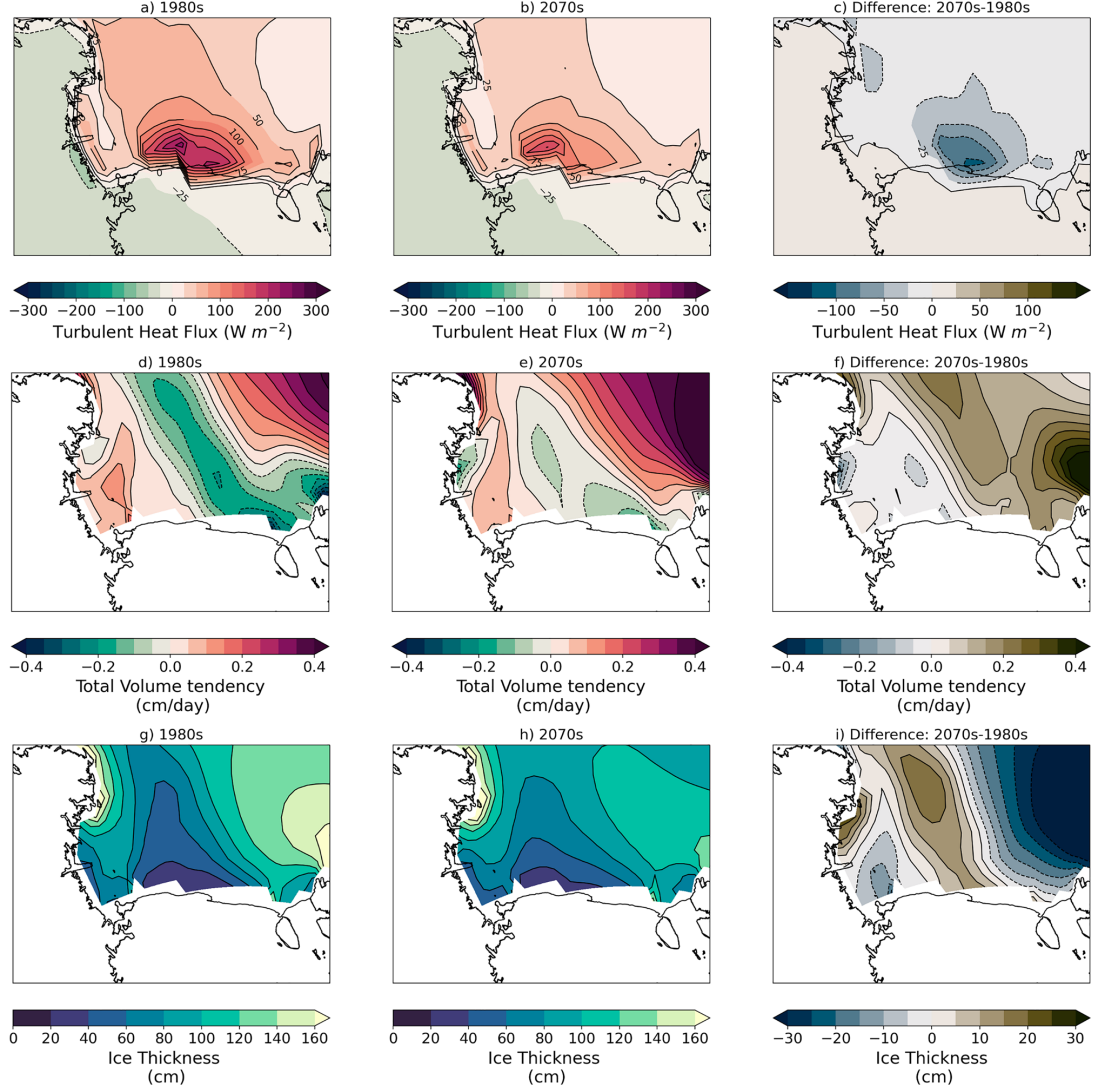
Decadal mean heat flux, sea ice volume tendency, and sea ice thickness for 1980s and 2070s. Total turbulent heat flux (W/m$^2$) for **a** 1980s, **b** 2070s, and **c** difference; total sea ice volume tendency (cm/day) for **d** 1980s, **e** 2070s, and **f** difference; sea ice thickness (cm) for **g** 1980s, **h** 2070s, and **i** difference. All differences are calculated as follows: Diff = (Pattern 0c − Pattern 2a). Positive (negative) heat flux values indicate atmospheric energy gain (loss). Positive (negative) ice tendency values indicate ice gain (loss)

We find that the differences in energy transfer to the atmosphere concentrated near the edge of the Ross Ice Shelf during winter in the 2070s are nearly 100 W/m$^{2}$ less than they were in the 1980s (Fig. 13a–c). The decadal turbulent flux differences are primarily driven by the sensible heat flux as we saw previously in pattern turbulent heat flux differences described in Sect. 3.1 (see Fig. 4). Additionally, we find that in the 2070s ice volume tendencies over the Ross Sea are more positive than they were in the 1980s (Fig. 13d–f). While there is a tongue of negative net ice volume tendencies in the 2070s, they are smaller in magnitude and less extensive than in the 1980s, which results in the total volume tendency over the Ross Sea south of 75$^\circ$ S latitude of − 0.28 km$^3$/day in the 1980s and $+$ 0.20 km$^3$/day in the 2070s. In the 2070s the net dynamic volume tendency (− 5.48 km$^3$/day) and thermodynamic volume tendency ($+$ 5.68 km$^3$/day) are actually higher in magnitude than the corresponding tendencies in 1980s (− 5.41 km$^3$/day and + 5.13 km$^3$/day, respectively). We also find that in the future there is a tongue of 10–15 cm (20%) thicker sea ice that extends northward from the Ross Ice Shelf into the Ross Sea (Fig. 13g–i). Thicker ice could lead to a delayed spring melt out and could delay primary productivity and could also impact the ice transport. One reason for the possible high volume transport in the 2070s despite weaker winds is that the ice is thicker in the Ross Sea so the net volume transport could increase. While the Ross Sea region has wintertime ice gain through thermodynamics in both decades, the dynamic loss exceeds gain in the 1980s making the region an ‘ice factory’, or net ice exporter of ice. By the 2070s, the thermodynamic ice gain exceeds dynamic loss and the region is no longer a net ice exporter.

**Fig. 14:**
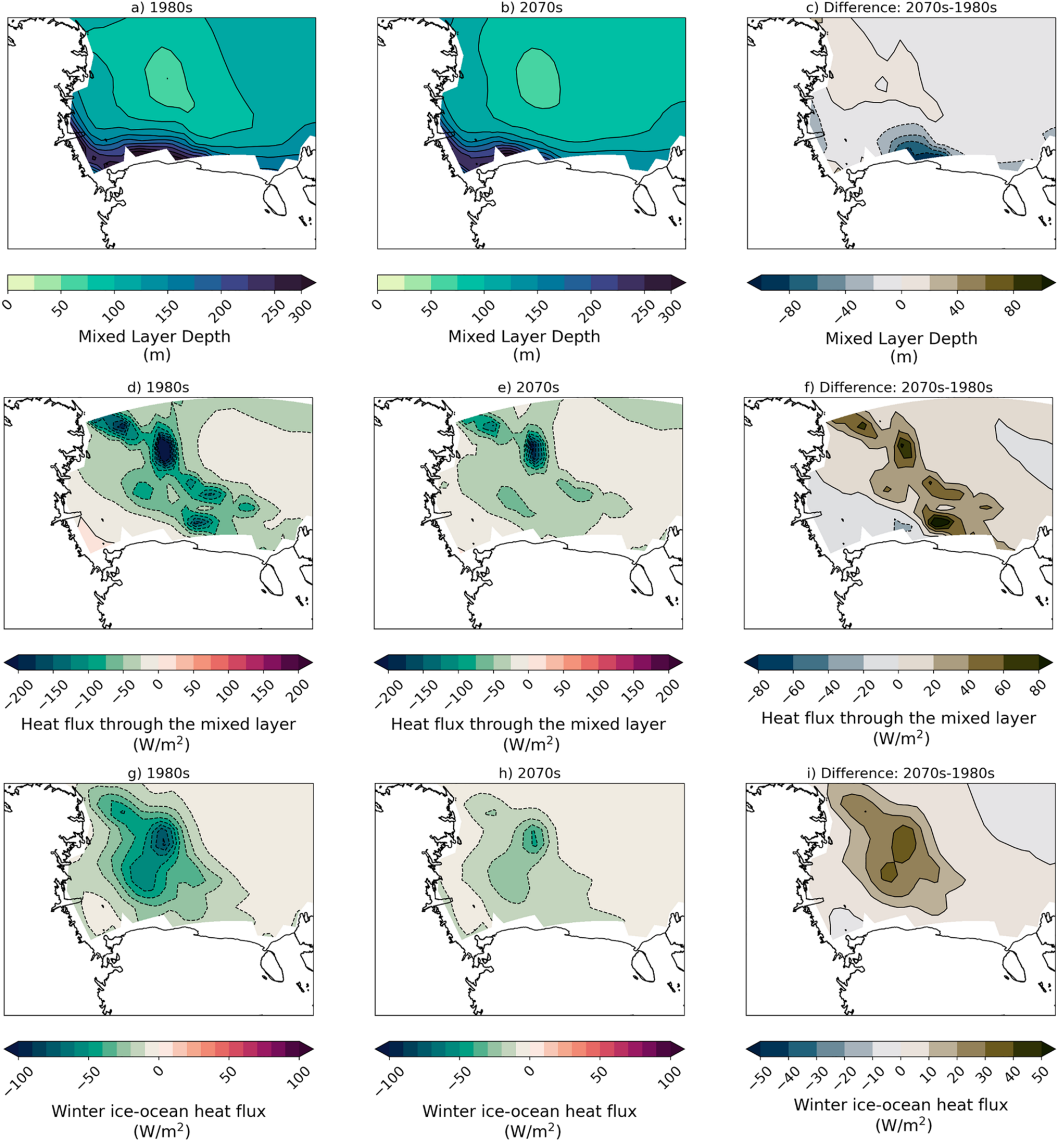
Decadal mean ocean mixing for 1980s and 2070s. Ocean mixed layer depth (m) for **a** 1980s, **b** 2070s, and **c** difference; heat flux through the mixed layer (W/m$^{2}$) for **d** 1980s, **e** 2070s, and **f** difference; ice–ocean heat flux (W/m$^{2}$) for **g** 1980s, **h** 2070s, and **i** difference. All differences are calculated as follows: Diff = (Pattern 0c − Pattern 2a). Negative heat flux through the mixed layer indicates an upward flux. Negative ice–ocean heat flux indicates ocean heat loss to the ice, usually due to sea ice melt

Greater magnitude thermodynamic ice growth in the 2070s despite the sharp decrease in polynya events also suggests that during polynya events feedbacks are likely at play that inhibit some of the thermodynamic growth. The end-of-winter (September) mixed layer depths in the ocean near the Ross Ice Shelf are 60 m (30%) shallower in the 2070s than the 1980s (Fig. 14a–c). As a result, the heat flux into the mixed layer due to entrainment of deeper waters decreases by up to 80 W/m$^{2}$ (Fig. 14d–f). This leads to decreases in the magnitude of the ice–ocean heat flux by 30 W/m$^{2}$ (50%), which indicates less ice melt from the ocean (Fig. 14g–i).

**Fig. 15:**
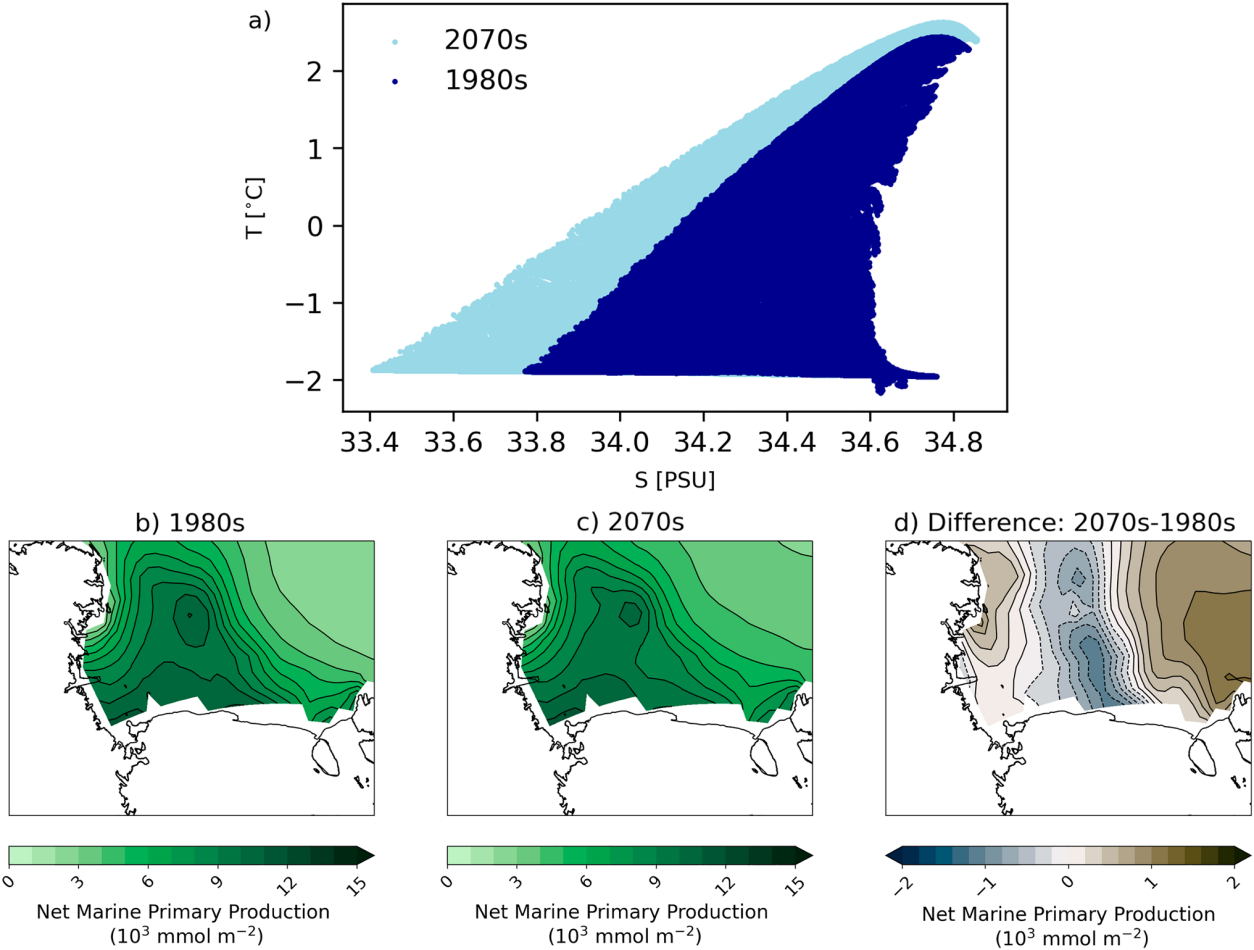
Decadal mean ocean variables for 1980s and 2070s. **a** Temperature-salinity diagram for all points in the Ross Sea at each depth between 0 and 600 m in the 1980s (dark blue) and 2070s (light blue). Net marine primary productivity (mmol/m$^{2}$) for **b** 1980s, **c** 2070s, and **d** difference. Differences are calculated as follows: Diff = (Pattern 0c − Pattern 2a)

We also find that over the top 600 m of the ocean in the Ross Sea region there is a general increase in temperature and decrease in salinity (Fig. 15a). A shift toward less dense water in this region suggests that there may be a decrease in AABW production during winter related to the decrease in ice production from polynya events. Fewer polynya events lead to less thermodynamic sea ice production, thus leading to less brine rejection and freshening of the water below. In addition to these possible impacts on AABW production, we also find changes in NPP. In the 2070s, in the central Ross Sea there is a decrease of up to 1 mmol/m$^{2}$ (10%) in subsequent growing season marine NPP (integrated over October to March), while there are increases in NPP elsewhere in the Ross Sea (Fig. 15b–d). The regions of decreased NPP originate at the Ross Ice Shelf where the polynya occurs, but extend farther north and closely correspond to the tongue of thicker ice in the 2070s (Fig. 13g–i). We propose that the decreases in NPP in the 2070s in the central Ross Sea are likely related to the thicker, more extensive sea ice that will take longer to melt the following spring and summer. Thus, due to increases in ice cover there may be less light available for primary production, however it is also possible that changes in the ocean mixing could impact nutrient availability that could also impact NPP.

## Discussion

This study assesses the biogeophysical conditions and changes in the Ross Sea during austral winter under anthropogenic climate change using the coupled CESM2-LE. To our knowledge this study is the first to use a coupled Earth system model to investigate projected changes in both physical and biological systems in the Ross Sea that may be relevant for both biologically protected regions and the global ocean circulation.

We use the SOM machine learning algorithm to identify sea ice patterns with CESM2-LE austral winter data. The SOM identifies sea ice patterns that range from total ice cover to large, polynya like patterns. Importantly, the SOM identifies these polynya patterns without any user determination of sea ice concentration or thickness thresholds, as has been a standard practice in past studies (e.g. Mohrmann et al 2021) but raise questions about how these thresholds are chosen. The SOM identifies a polynya feature near the Ross Ice Shelf that is comparable in location and size to the large, well-known Ross Sea Polynya (Arrigo and van Dijken 2003; Tamura et al 2016). The relatively small Terra Nova Bay Polynya that occurs on the west coast of the Ross Sea (Arrigo and van Dijken 2003; Tamura et al 2016) is not identified by the SOM in any pattern. The inability for CESM2 to clearly produce a polynya in Terra Nova Bay is likely related to both the small scale of the feature and coarse CESM2 model grid and missing processes in CESM2 related to ice shelves or ice tongues that are uniquely important for the Terra Nova Bay Polynya formation (Bromwich and Kurtz 1984). Despite not identifying the Terra Nova Bay Polynya, we are able to discern significant biogeophysical changes in the Ross Sea Polynya under future climate change.

Using the SOM patterns, we find that in addition to lower sea ice concentrations associated with polynya events, (Fig. 2) there is also anomalously thinner sea ice over most of the Ross Sea (Fig. 3). As a result of more open ocean and thinner ice, there are large turbulent heat fluxes (300+ W/m$^{2}$) into the atmosphere during polynya events that are primarily due to large sensible heat fluxes (Fig. 4). There are few observations of turbulent fluxes over polynyas during winter, but two campaigns have measured fluxes over the Terra Nova Bay polynya in winter and show that they can be extremely large (500–2000 W/m$^{2}$), though highly spatially variable, and dominated by the sensible heat flux (Knuth and Cassano 2014; Ackley et al 2020). Attempting to compare the CESM2-LE polynya fluxes with the observations highlights several items of interest. First, the CESM2-LE fluxes are daily average fluxes, so they are likely to be lower than instantaneous in-situ fluxes. Second, while the CESM2-LE fluxes are elevated during the polynya events they are likely still lower than observations because the coarse model grid will not be able to well resolve small scale sea ice features or the intense wind speeds that drive polynyas and large fluxes. While the CESM2-LE fluxes are lower than observations, we are encouraged that the CESM2-LE captures the relatively large turbulent heat fluxes over polynyas and the partitioning between sensible and latent heat during these events.

Transfer of energy from the open ocean to the atmosphere during polynya events leads to ocean cooling and eventually sea ice formation. In CESM2-LE, polynya events lead to a net export of sea ice out of the Ross Sea (− 1.08 km$^3$/day south of 75$^\circ$ S latitude), primarily due to the large dynamic ice volume loss as ice is advected northward. But there is also elevated thermodynamic ice growth during polynya events (6+ cm/day) along the Ross Ice Shelf compared to the non-polynya events (3 cm/day) (Fig. 5). Elevated ice production in CESM2-LE during polynya events is also consistent with satellite and in-situ estimates of ice production. Satellite based methods suggest sea ice production rates of 6–13 cm/day in polynyas (Tamura et al 2016), while in-situ estimates in Terra Nova Bay range from daily average rates of 15–30 cm/day (Schick 2018; Thompson et al 2020) to instantaneous rates of ~ 70–100 cm/day (Ackley et al 2020; Thompson et al 2020). As with the turbulent heat fluxes, the lower thermodynamic ice production in CESM2-LE compared to observational methods is not surprising given the coarse grid and the daily average timescale. Additionally, it is likely that CESM2-LE is missing critical ice formation processes under strong wind conditions (e.g. rapid formation and rafting of pancake ice) or environmental conditions (e.g. ice tongues) that may impact model estimates of sea ice formation as compared to observational estimates. While thermodynamic ice production in the model is higher during polynya events (+ 5.46 km$^3$/day) compared non-polynya events (+ 5.07 km$^3$/day), we believe the reason it is not more elevated is that feedbacks with the ocean, such as polynya events driving deeper ocean mixing that brings ocean heat upward, may inhibit sea ice growth (Fig. 6). Given that the aim of this paper is to assess how polynya events change in the future within the Ross Sea, future work is needed to fully understand the processes during polynya events in the model and to discern if any crucial processes are missing.

A novel finding that this study reveals is that in the future winter sea ice concentrations are projected to increase in the southern Ross Sea despite general increases in temperature from warming. These results are from a single model, and a reasonable question is whether we should trust CESM2-LE projections in winter sea ice cover in the southern Ross Sea given the inability for most coupled climate models to capture observed Antarctic-wide sea ice concentration trends, (Turner et al 2015; Shu et al 2020; Casagrande et al 2023). Given that studies have shown there is large internal climate variability in Antarctic sea ice concentration and that observed positive trends may be within modeled variability, we do not believe that models should be excluded because they have pan-Antarctic negative trends (Turner et al 2015; Roach et al 2020). Additionally, the largest discrepancies in sea ice concentration trends between models and observations are in austral autumn and this study focuses on austral winter when discrepancies are smaller. Recent experiments with nudged winds, sea ice drift, and/or sea surface temperatures near the sea ice edge have been able to replicate the observed positive trends in Antarctic sea ice concentration (Sun and Eisenman 2021; Blanchard-Wrigglesworth et al 2021). The ability for coupled climate models to replicate observed trends when correcting ice drift near the ice edge suggests that errors in the ice trends may be primarily related to ice processes near the northward ice edge rather than fundamental problems with ice production near the Antarctic coast. Indeed, in CESM2-LE the decline in winter sea ice concentration in the Ross Sector of the Antarctic is due to large declines at the northern ice edge (Fig. 1). In the southern Ross Sea, the sea ice concentration trend is positive primarily because in the future polynya events are likely to become less frequent (Fig. 8, which leads to a larger area that has high ice concentrations. It should be noted that most climate models, including CESM2-LE do not have interactive ice shelf melt in response to future warming. Recent observational studies have found that the Ross Sea is freshening due to ice shelf melt (Jacobs et al 2022), and the ice shelf melt not only affects salinity but also the nutrients necessary for primary productivity (Arrigo et al 2015). Model experiments that include freshwater input from ice shelf melt (e.g. Bintanja et al 2013; Pauling et al 2016, 2017; Moorman et al 2020) have variable results in terms of the impact on sea ice trends that can also differ regionally. If prognostic ice shelf melt and nutrient fluxes associated with that melt were included in the CESM2-LE experiments it is likely that sea ice and productivity trends would be impacted. However, the precise ways in which it might change are unknown and not accounted for in this study. Future work with prognostic ice shelf freshwater and nutrient fluxes is needed to better explore these influences (e.g. Pattyn 2018; Muntjewerf et al 2021).

We use frequency with which SOM training data were mapped to each pattern to determine that the decrease in polynya events is responsible for ~ 90% of the sea ice concentration difference from the 1980s to the 2070s (Fig. 7). We find that in the location where sea ice concentrations decrease most in future decades there are corresponding decreases in wind speed (Fig. 9), likely driven by a combination of large scale circulation changes related to the strength and position of the Amundsen Sea Low and stability and surface roughness local positive feedbacks that further decrease near-surface wind speeds (Figs. 10, 11, 12). The relationship between ocean mixing and polynyas may also be a positive feedback in this case where reduced ocean mixing leads to a reduced ocean-ice heat flux and would allow for more sea ice growth in winter. While it is outside the scope of this present study, future work could extend to other coupled models and other seasons.

Finally, we investigate the impacts of winter polynyas and sea ice on biology and water mass properties in future projections. We find that the water from 0 to 600 m in the Ross Sea are warming and freshening in the 2070s compared to the 1980s, which would lead to less dense water and a possible decline in AABW formation in the future. A future decrease in Ross Sea AABW production is consistent with recent studies in the Ross Sea and Weddell Sea that have shown periods of decreasing AABW density related to climate (Silvano et al 2020; Zhou et al 2023), though there has been a small rebound in Ross Sea AABW density due to increasing salinity since 2014 (Castagno et al 2019; Silvano et al 2020). AABW production and water properties experience long term variability that may make a significant trend difficult to discern. While this study does not focus on changes in AABW, further investigation of these changes and related processes is important because it has the capacity to impact the global ocean circulation. As regions of lower sea ice concentration and thickness, polynyas may also precondition biological productivity as they experience earlier ice retreat than surrounding areas or enhanced mixing of nutrients. While it is beyond the scope of this study to fully investigate the biological community composition changes and limitation terms, we do investigate the relationships between winter polynya occurrence and net primary productivity the following summer. While we find relatively weak relationships between the frequency of polynya events and NPP in the following summer (Fig. 6), we do find stronger relationships between polynya event frequency and ocean mixing and ice–ocean heat fluxes that same winter. Additionally, by comparing the NPP in the 1980s and 2070s we find that there is approximately ~ 10% decrease in NPP in the center of the Ross Sea (Fig. 15) that corresponds to more extensive and thicker winter ice conditions (Fig. 13). This correspondence suggests that as the sea ice conditions change they may affect some of the physical conditions relevant for NPP in the Ross Sea. The relevant future processes that could impact declining NPP might be light availability as thicker and more extensive ice could delay ice retreat the following summer, or through ocean mixing impacts on nutrient availability. A full assessment of the modeled NPP, including annual cycles, limiting terms, etc. are beyond the scope of this manuscript but could be relevant for detailed projections of the biogeochemisty in the Ross Sea. It is worth noting that reduction of NPP may propagate up the food chain and could have larger impacts on the entire Ross Sea ecosystem. As the Ross Sea is one of the few locations around Antarctica where Emperor Penguins, a threatened species, may be able to survive in future climate scenarios (Jenouvrier et al 2014, 2019), it is especially relevant to understand the relationships between physical and biological systems in this region that could impact species that the Ross MPA is intended to protect. Both male and female Emperor Penguins require access to open water throughout the winter months to feed at sea (Jouventin and Dobson 2018). All Emperor Penguin colonies in the Ross Sea are located near polynyas that remain open in the winter (Kooyman 1993), and in the last glacial maximum when sea ice was more extensive the conditions were sub-optimal for Emperor Penguins (Younger et al 2015). Further studies to better understand connections between Emperor Penguin populations and sea ice conditions could help elucidate declining winter polynya events may impact the penguin demographics in the Ross Sea. Additionally, future analysis could be performed in other Antarctic regions where there are sensitive ecosystems to investigate whether there are similar relationships between sea ice and biology.

## Conclusions

This study is a novel investigation of changing winter sea ice conditions in the southern Ross Sea and their biogeophysical impacts in the region using the CESM2-LE. We focus on the austral winter (July-August-September) and use the SOM machine learning algorithm to answer the following questions:

*What wintertime sea ice concentration patterns exist in the Ross Sea, and how and why are these patterns changing in time?* The SOM identifies winter sea ice patterns from CESM2-LE that include a large Ross Sea Polynya, but it is unable to capture the smaller Terra Nova Bay Polynya. We find that the frequency of Ross Sea Polynya patterns declines sharply in future decades. The decline in polynyas is likely initiated by wind speed decreases that decrease advection of ice away from the southern Ross Sea. The decline in wind speed is likely due to large scale atmospheric circulation changes related to the strength and position of the Amundsen Sea Low. Declines in wind would then initiate local positive feedbacks that would further decrease wind speed: increasing local atmospheric stability and increasing surface roughness, both of which could lead to decreases in near-surface wind speed. Additionally, a positive feedback where decreased ocean mixing during polynya events could reduce the ocean-ice heat flux could also lead to increasing sea ice cover in this region.

*What are the biogeophysical implications of the winter sea ice cover in the Ross Sea?* During polynya events there are enhanced heat fluxes from the surface to the atmosphere which drive ice production during a polynya event, and the sea ice is advected northward. In the future, as polynya events decline, so do turbulent heat fluxes and the future Ross Sea is no longer a ‘sea ice factory‘ that exports ice northward. Ocean mixed layer depth decreases in the future when polynya events decline, which could impact the ocean-ice heat flux, AABW production, and phytoplankton. The relationship between the frequency of wintertime polynya events and NPP is relatively weak, but there may be a stronger relationship in the future as decreasing polynya frequency drive thicker, more extensive ice that will impact light availability for production. Ultimately, better understanding these biogeophysical relationships will be important for assessing how the Ross Sea ecosystem, which is currently protected by an MPA, may be vulnerable in the future in unexpected ways and how water mass formation in this region may impact the global ocean circulation.

## Supplementary Information

Below is the link to the electronic supplementary material.

Supplementary file1 (PDF 12,067 KB)

## Data Availability

Previous and current CESM versions are freely available online at the CESM2 website: https://www.cesm.ucar.edu/models/cesm2/. The CESM2-LE data used in this study are freely available the CESM2-LE website: https://www.cesm.ucar.edu/projects/community-projects/LENS2/.

## Code availability

Jupyter Notebooks used for the Self Organizing Map training and the data analysis are available at the following GitHub Repository: https://github.com/duvivier/CESM2_Ross_Sea_polynya_analysis.
