# Supplementary material for: Projections of winter polynyas and their biophysical impacts in the Ross Sea Antarctica
Source: Clim Dyn. 2023 Sep 23;62(2):989–1012. doi: 10.1007/s00382-023-06951-z (PMC11424701; doi:10.1007/s00382-023-06951-z)
Supplement: Supplementary file 1 — Supplementary file1 (PDF 12,067 KB) [file 382_2023_6951_MOESM1_ESM.pdf]

Projections of Winter Polynyas and Their Biophysical Impacts in the Ross Sea Antarctica

Alice K. DuVivier[1*], Maria J. Molina[2,1], Anna-Lena Deppenmeier[1], Marika M. Holland[1], Laura Landrum[1], Kristen Krumhardt[1] and Stephanie Jenouvrier[3]

[1*]Climate and Global Dynamics, National Center for Atmospheric Research, Boulder, CO, USA.
[2]Department of Atmospheric and Oceanic Science, University of Maryland, College Park, MD, USA.
[3]Woods Hole Oceanographic Institution, Falmouth, MA, USA.

*Corresponding author(s). E-mail(s): duvivier@ucar.edu;

1 Description

This supplementary information contains 17 figures that provide additional information for the manuscript listed above, but are not essential for the analysis presented.

Supplementary Figures

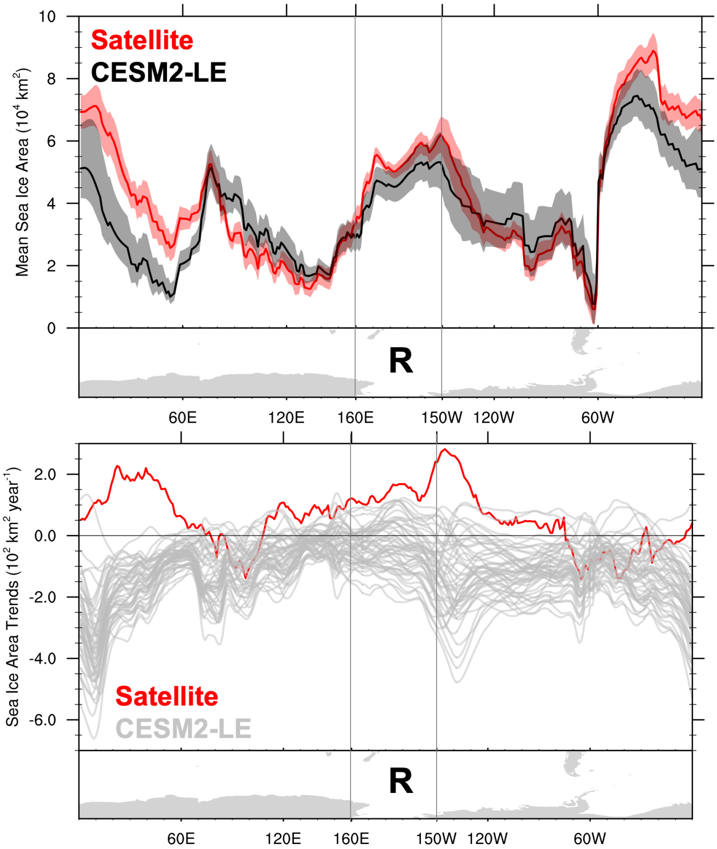

Fig. S1 Top panel shows mean Antarctic winter (JAS) sea ice area (+/- one standard deviation) by longitude from 1979-2014 from NSIDC CDR satellite observations (red) and the CESM2-LE (black). Bottom panel shows the 36 year sea ice area trends over 1979-2014 by longitude from NSIDC CDR satellite observations (red) and for all 50 members of the CESM2-LE (grey) that use standard CMIP6 forcing. The Ross Sea sector is marked by vertical lines and labeled with an 'R'.

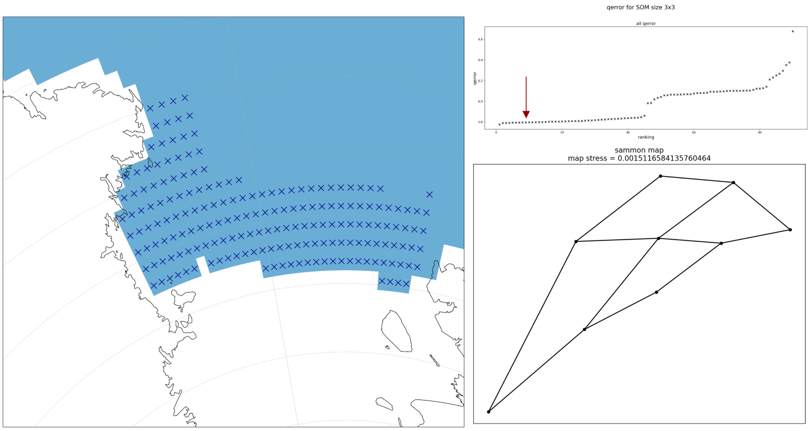

Fig. S2 Ice grid points in the southern Ross Sea that were used in training the SOM are shown as 'X's on the map in panel a. The error for the winning SOM combination is shown by the arrow in panel b. The sammon map for the winning SOM combination is shown in panel c. Training: daily ice concentration. 3x3 with parameters: sig=1.0, lr=0.005, iter=100,000

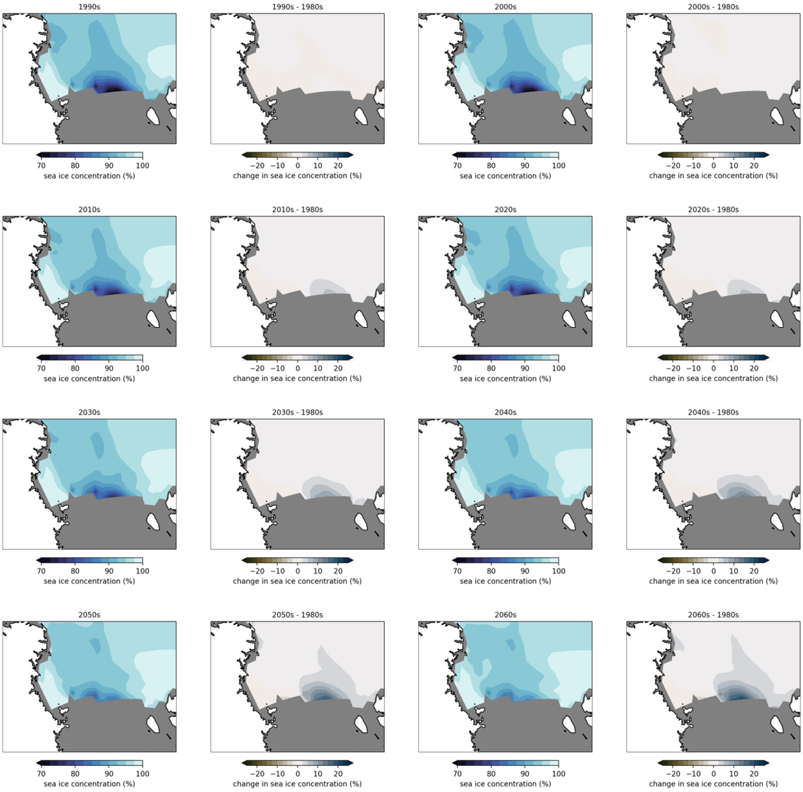

Fig. S3 Winter sea ice concentration (%) in the Ross Sea for each decade 1990s-2070s (blue shading) and difference from 1980s (brown and blue shading).

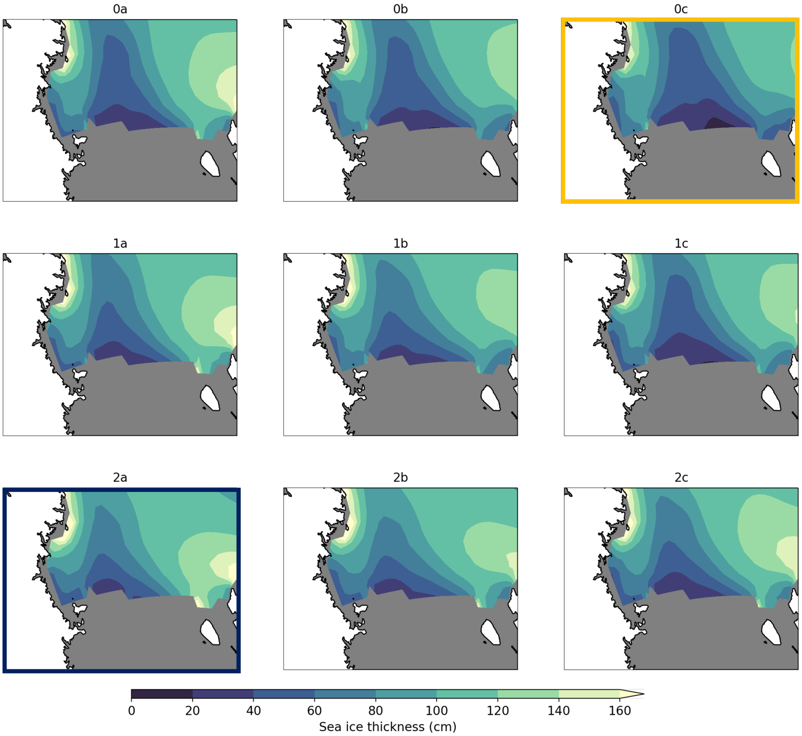

Fig. S4 Composite sea ice thickness (cm) for each of the nine patterns identified by the SOM. Pattern 0c (gold border) and 2a (dark blue border) indicate the patterns that will be analyzed in more detail.

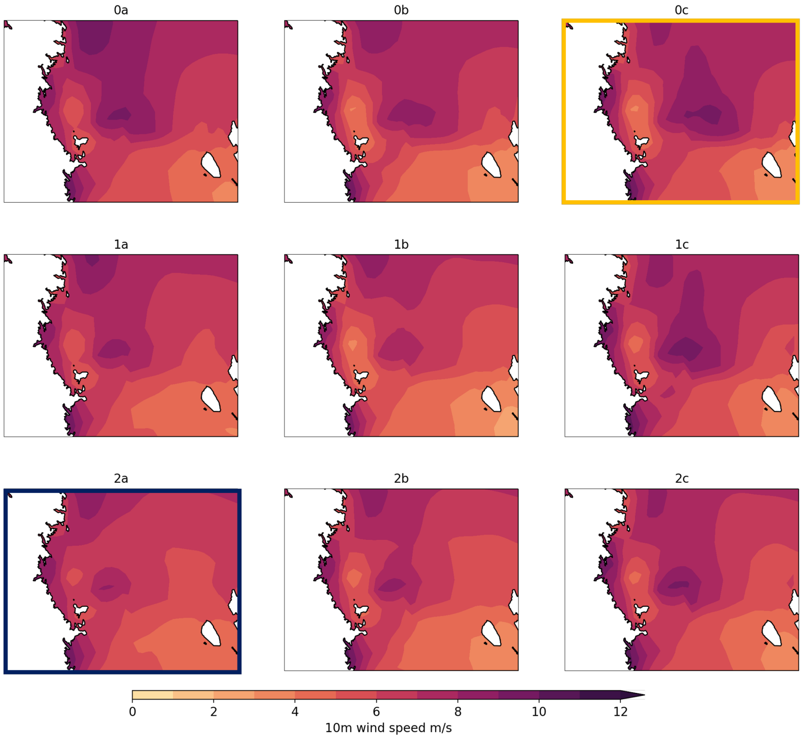

Fig. S5 Composite wind speed (m/s) for each of the nine patterns identified by the SOM. Pattern 0c (gold border) and 2a (dark blue border) indicate the patterns that will be analyzed in more detail.

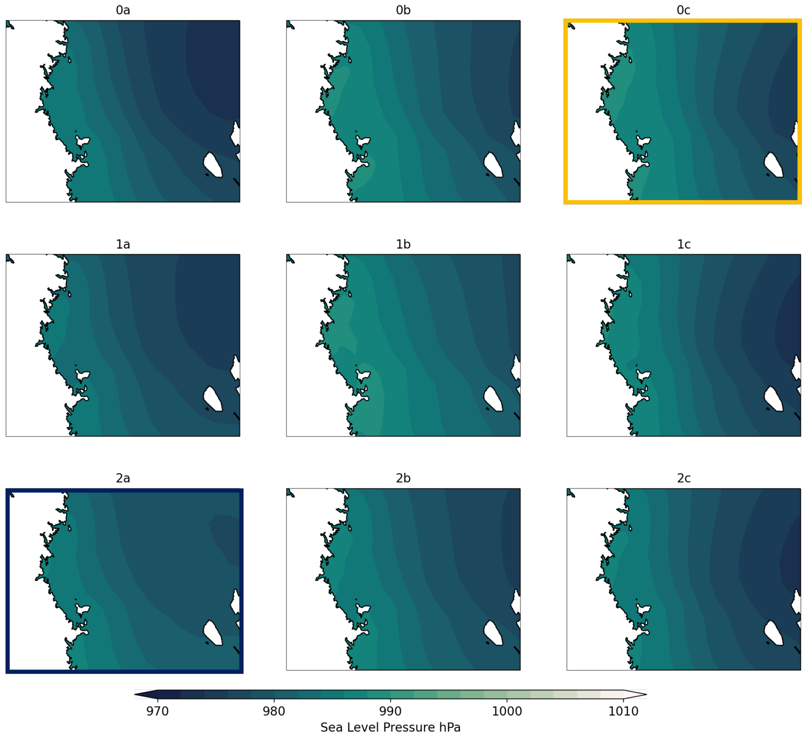

Fig. S6 Composite mean sea level pressure (hPa) for each of the nine patterns identified by the SOM. Pattern 0c (gold border) and 2a (dark blue border) indicate the patterns that will be analyzed in more detail.

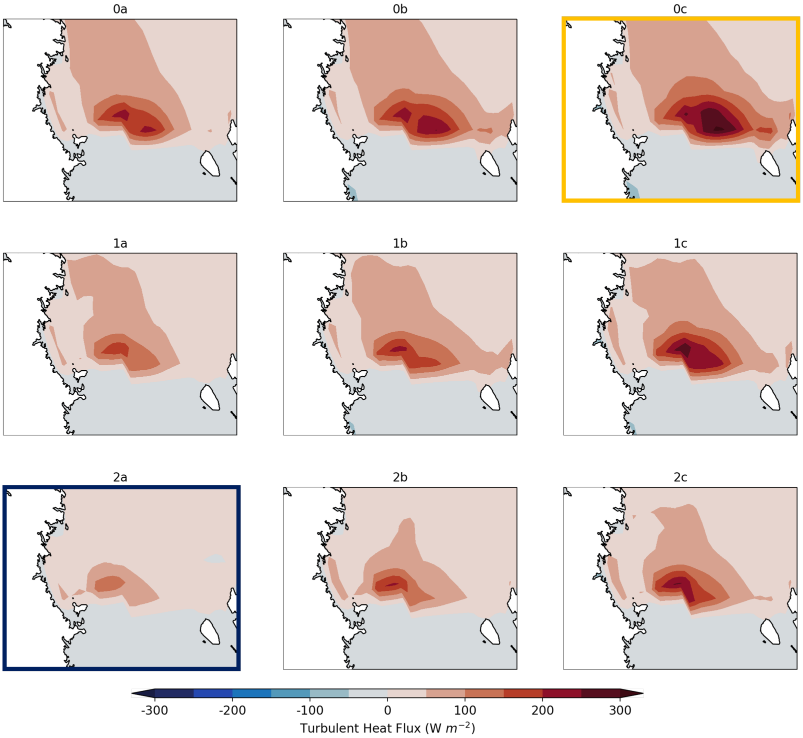

Fig. S7 Composite mean total turbulent heat flux (W/m^2) for each of the nine patterns identified by the SOM. Pattern 0c (gold border) and 2a (dark blue border) indicate the patterns that will be analyzed in more detail.

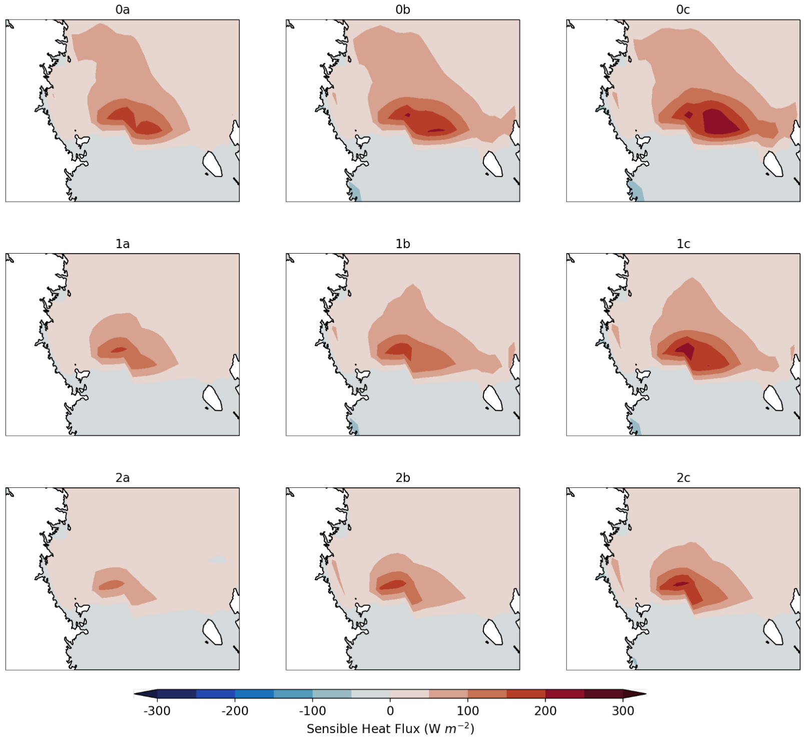

Fig. S8 Composite mean sensible heat flux (W/m^2) for each of the nine patterns identified by the SOM. Pattern 0c (gold border) and 2a (dark blue border) indicate the patterns that will be analyzed in more detail.

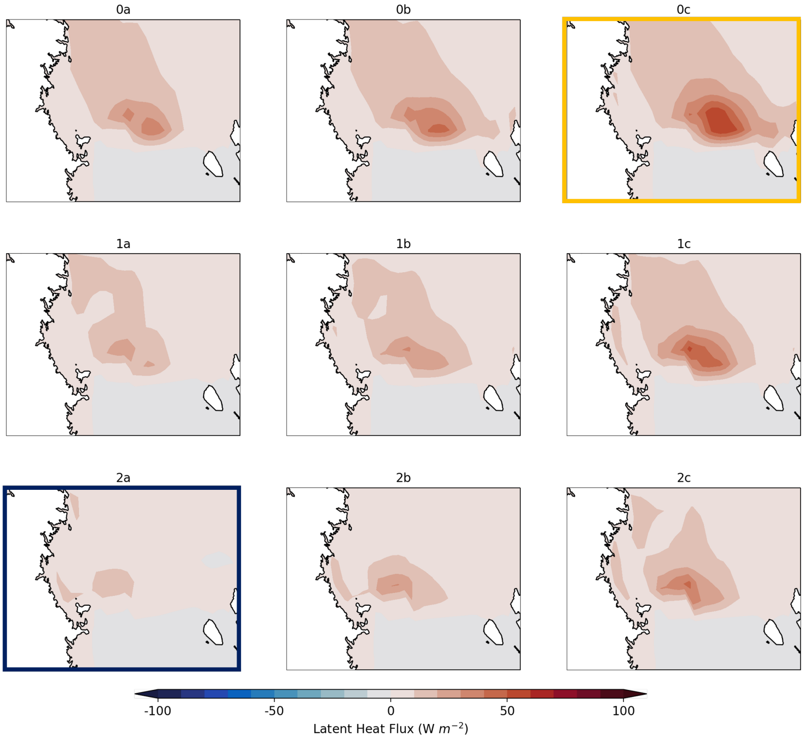

Fig. S9 Composite mean latent heat flux (W/m^2) for each of the nine patterns identified by the SOM. Pattern 0c (gold border) and 2a (dark blue border) indicate the patterns that will be analyzed in more detail.

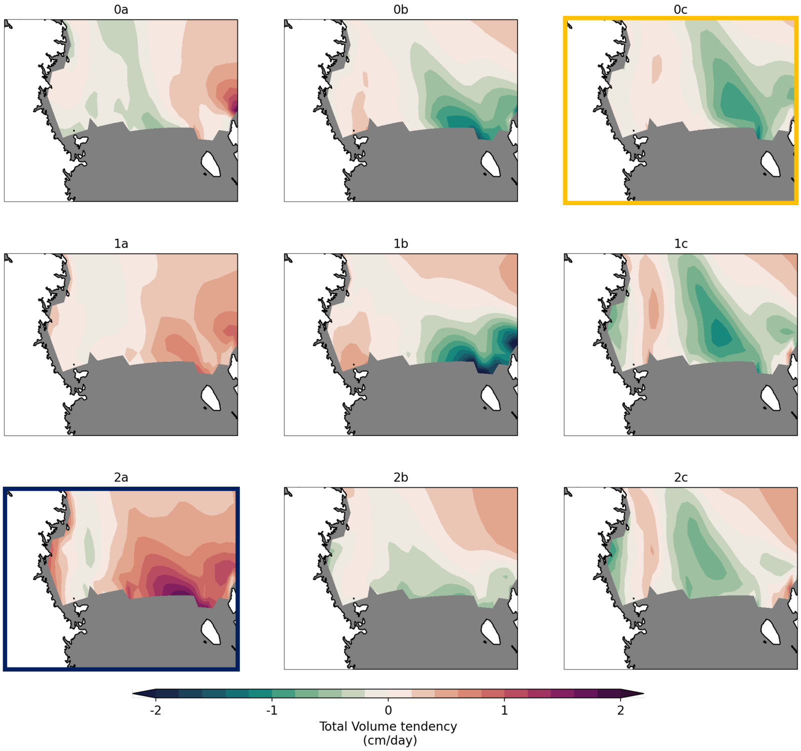

Fig. S10 Composite mean total sea ice volume tendency (cm/day) for each of the nine patterns identified by the SOM. Pattern 0c (gold border) and 2a (dark blue border) indicate the patterns that will be analyzed in more detail.

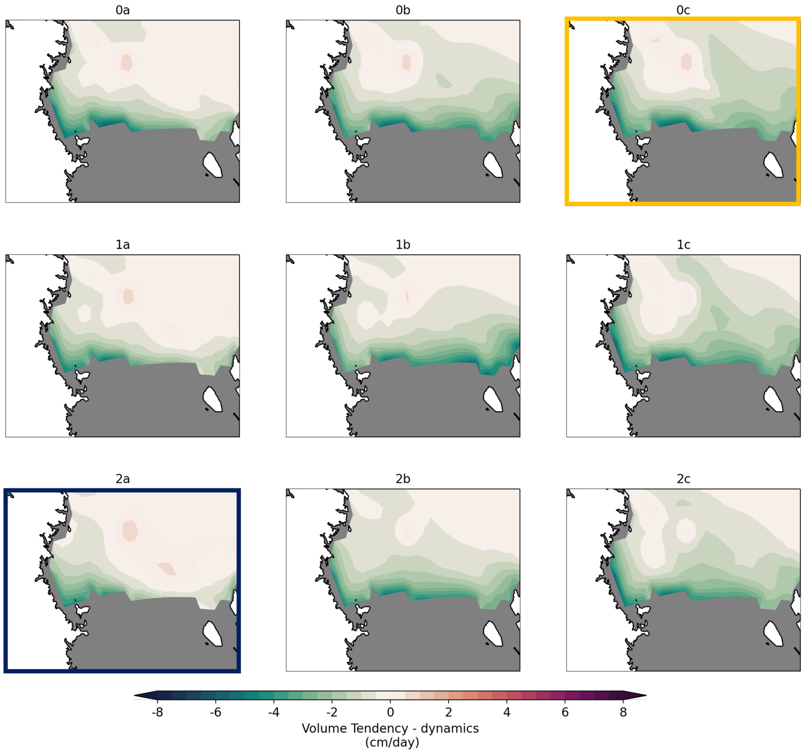

Fig. S11 Composite mean dynamic contribution to the total sea ice volume tendency (cm/day) for each of the nine patterns identified by the SOM. Pattern 0c (gold border) and 2a (dark blue border) indicate the patterns that will be analyzed in more detail.

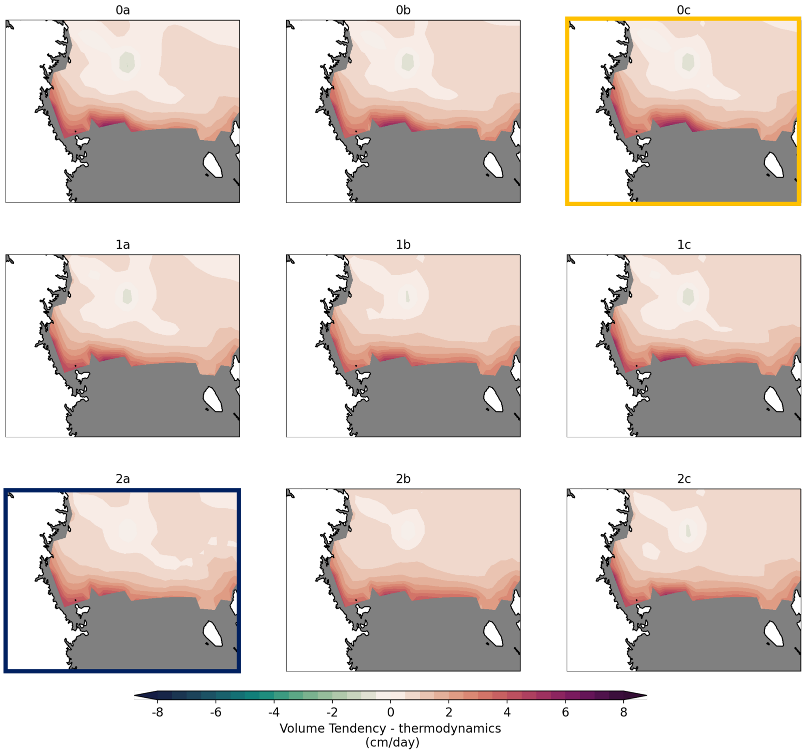

Fig. S12 Composite mean thermodynamic contribution to the total sea ice volume tendency (cm/day) for each of the nine patterns identified by the SOM. Pattern 0c (gold border) and 2a (dark blue border) indicate the patterns that will be analyzed in more detail.

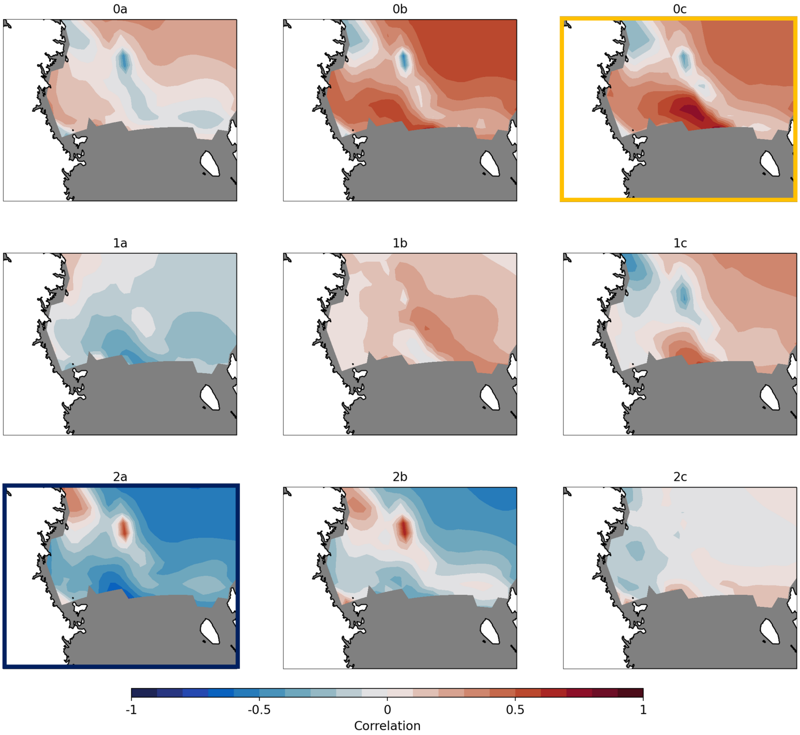

Fig. S13 Correlation of end of winter (September) ocean mixed layer depth with SOM pattern frequencies. Pattern 0c (gold border) and 2a (dark blue border) indicate the patterns that will be analyzed in more detail.

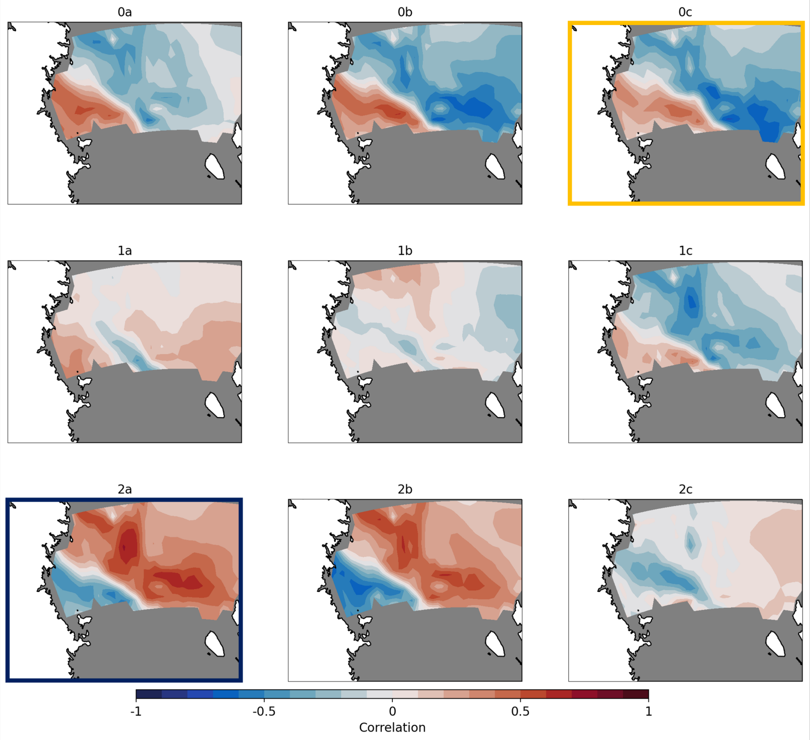

Fig. S14 Correlation of end of winter (September) heat flux at the ocean mixed layer depth with SOM pattern frequencies. Positive heat fluxes are downward. Pattern 0c (gold border) and 2a (dark blue border) indicate the patterns that will be analyzed in more detail.

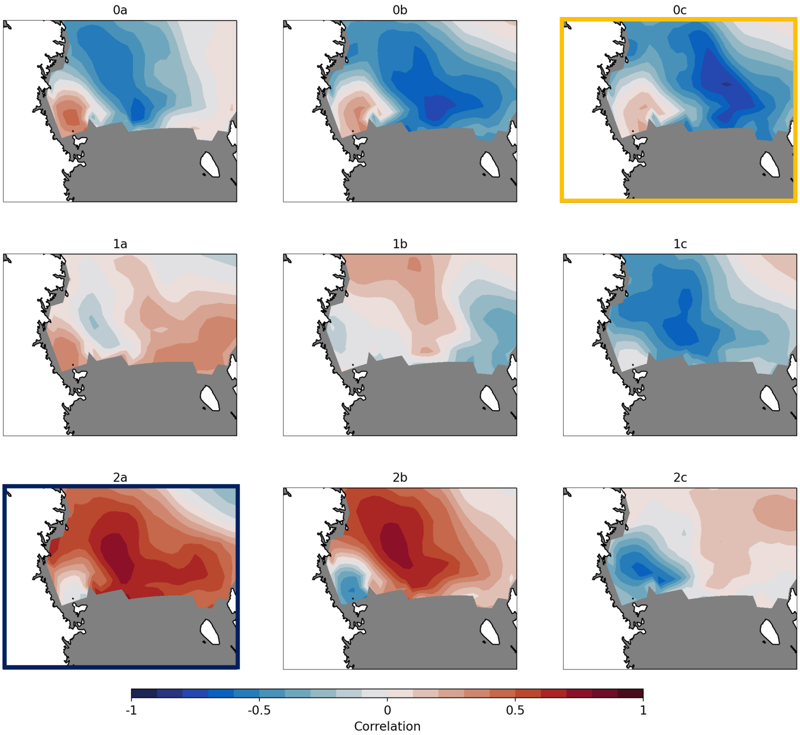

Fig. S15 Correlation of winter mean heat flux from the ocean to ice with SOM pattern frequencies. Positive heat fluxes indicate heat transfer from the ice to the ocean (through freezing). Pattern 0c (gold border) and 2a (dark blue border) indicate the patterns that will be analyzed in more detail.

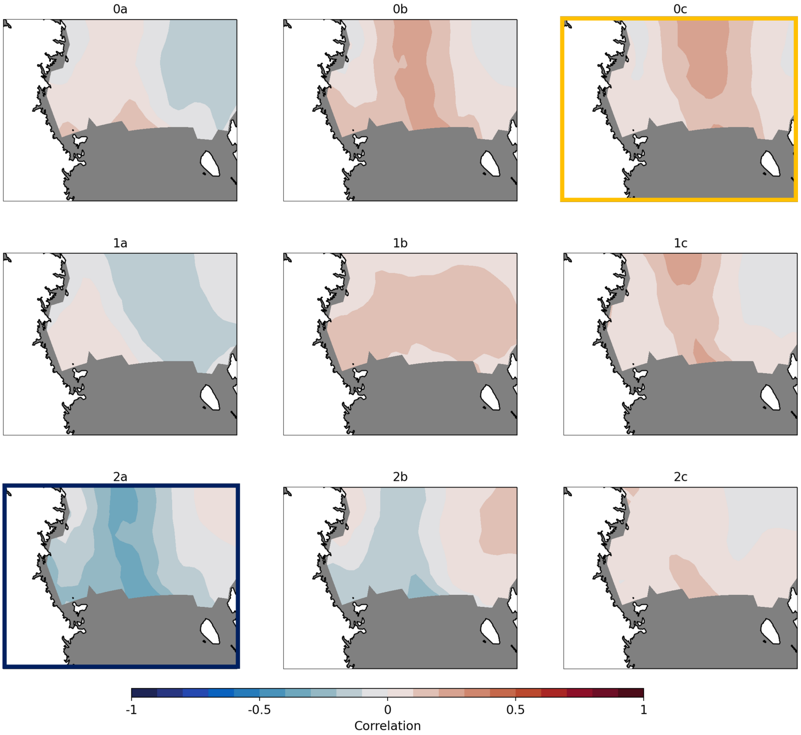

Fig. S16 Correlation of following summer total marine net primary productivity with SOM pattern frequencies. Pattern 0c (gold border) and 2a (dark blue border) indicate the patterns that will be analyzed in more detail.

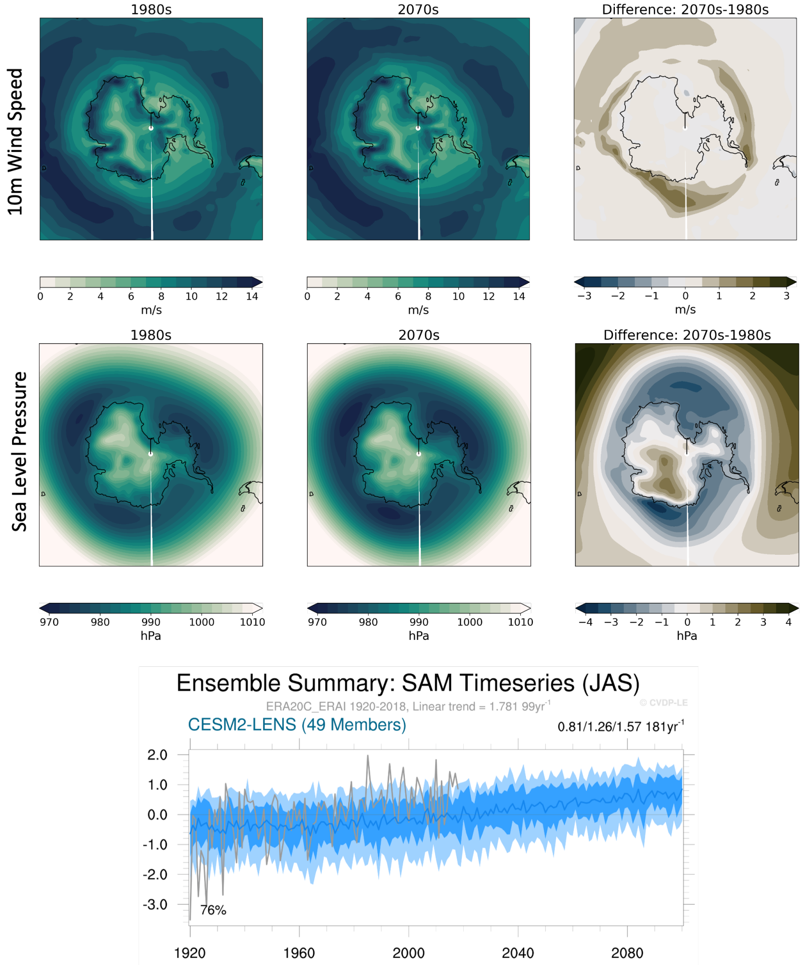

Fig. S17 Mean winter wind speed and sea level pressure and difference 1980s and 2070s. A timeseries of the Southern Annular Mode (SAM) from the CESM2-LE and reanalysis is shown below the maps.
